# A Selective MAP3K1 Inhibitor Facilitates Discovery of NPM1 as a Member of the Network

**DOI:** 10.3390/molecules30092001

**Published:** 2025-04-30

**Authors:** Lidia Boghean, Sarbjit Singh, Kiran K. Mangalaparthi, Smitha Kizhake, Lelisse Umeta, Donn Wishka, Paul Grothaus, Akhilesh Pandey, Amarnath Natarajan

**Affiliations:** 1 Eppley Institute for Research in Cancer and Allied Diseases, University of Nebraska Medical Center, Omaha, NE 68198, USA; 2 Department of Laboratory Medicine and Pathology, Center for Individualized Medicine, Mayo Clinic, Rochester, MN 55905, USA; mangalaparthi.kiran@mayo.edu (K.K.M.); pandey.akhilesh@mayo.edu (A.P.); 3 Drug Synthesis and Chemistry Branch, National Cancer Institute, NIH, Bethesda, MD 20892, USAgrothausp@mail.nih.gov (P.G.); 4 Department of Community Medicine, Manipal Academy of Higher Education, Manipal 576104, India; 5 Fred and Pamela Buffett Cancer Center, University of Nebraska Medical Center, Omaha, NE 68198, USA

**Keywords:** MAP3K1 inhibitor, quinoxaline, phosphoproteomics, NPM1

## Abstract

The quinoxaline core is found in several biologically active compounds, with Erdafitinib being the first FDA-approved quinoxaline derivative that targets a kinase and exhibits anti-cancer properties. We previously reported a quinoxaline analog (**84**) that displayed anti-cancer effects by inhibiting IKKβ, a key kinase in the NFκB pathway. Here, we present the synthesis of a regioisomer (**51**-**106**) and its characterization as a selective MAP3K1 inhibitor with improved metabolic stability and oral bioavailability. We used the small molecule MAP3K1 inhibitor in a proteomics study that identified NPM1 as a member of the MAP3K1 network.

## 1. Introduction

The mitogen-activated protein kinase (MAPK) pathway is a cascade of three kinases that sequentially phosphorylate and activate their substrates. The 24 mitogen-activated kinase kinase kinases (MAP3Ks) are rapidly activated fibrosarcoma (RAF1), BRAF, ARAF, and 21 MAP3K (1–21) that phosphorylate mitogen-activated kinase kinases (MAP2Ks), which in turn phosphorylate MAPKs to regulate an array of cellular processes, including proliferation and differentiation [1]. MAP3K1 is a 1512 amino acid protein with two zinc finger domains (SWIM-type^338-366^ and RING-type^443-492^), a tumor overexpressed gene (TOG^542-888^) domain, and a kinase domain (1243–1508) at its C-terminus [2]. The complexity of the MAP3K1-associated signaling network is highlighted by the differential response to cellular cues that promote signaling through an array of downstream effectors such as extracellular signal-related kinase (ERK) [3,4], c-jun N-terminal kinase (JNK) [5,6,7,8,9], p38 [10,11], and nuclear factor kappa-light-chain-enhancer of activated B cells (NFκB) [12,13,14]. Thus, there is a need for small molecule probes that selectively and reversibly perturb MAP3K1 to dissect the associated signaling network.

Pancreatic cancer is a disease with a low 5-year survival. In pancreatic cancer patients, elevated levels of MAP3K1 have been associated with lymph node metastasis [15], and its overexpression is associated with poor survival [16]. While novel therapeutic strategies continue to be evaluated in clinical trials, gemcitabine, a nucleoside that targets DNA synthesis, remains a main chemotherapeutic agent for pancreatic cancer. MAP3K1 has been suggested to play a pro-survival role in the DNA damage response, and DNA damage-induced cell death is increased when MAP3K1 is knocked down [17]. MAP3K1 kinase function has also been implicated in the growth, invasion, and migration of pancreatic cancer cells and expression of kinase-dead, MAP3K1-promoted cell death in pancreatic cancer cell lines [16]. Thus, a selective MAP3K1 inhibitor has the potential to be developed as a pancreatic cancer therapeutic.

We have had a long-standing interest in exploiting the differential π electron density among the three set of carbon atoms on the quinoxaline core to generate derivatives and explore their anticancer effects. Subjecting quinoxaline analog libraries to both unbiased and biased screening strategies identified an orally bioavailable, non-ATP competitive inhibitor of nuclear factor kappa-B kinase subunit beta (IKKβ) inhibitor that exhibited anti-cancer effects in mouse models [18,19,20,21,22,23,24]. A structure–activity relationship (SAR) study guided by a cell-based reporter assay that specifically reads out tumor necrosis factor (TNF) α-induced IKKβ-mediated NFκB activation identified analog **84**, which exhibited ~4-fold and ~5.7-fold improved potency and bioavailability, respectively [25]. Analog **84** inhibited tumor growth by itself and in combination with gemcitabine in a pancreatic cancer mouse model. Despite the remarkable activity, the low bioavailability (F = 17.6%) of **84** did not meet the industry minimum of ≥30% for a candidate to advance with manageable risk [26].

In the present study, we hypothesized that repositioning the F atom to block a site of metabolic vulnerability in analog **84** would improve metabolic stability and bioavailability. To test this, we synthesized the regioisomer (**51**-**106**) [27], investigated its effects on MAP3K1-IKKβ-NF-κB signaling, and used it as a small molecule tool to investigate global phosphorylation events associated with MAP3K1 inhibition. Phosphoproteomic analyses following **51**-**106** treatment revealed that nucleophosmin (NPM1)^T199^ phosphorylation is decreased upon MAP3K1 inhibition and is associated with cell cycle regulation in pancreatic cancer cells. NPM1 is a multifaceted protein implicated in various cellular processes, including ribosome assembly [28,29,30], mRNA processing [31], DNA duplication [32,33,34,35], nucleocytoplasmic protein trafficking [36,37], cell cycle progression, and the DNA damage response [38,39,40,41,42,43]. This study suggests that NPM1 is part of the MAP3K1 network.

## 2. Results and Discussion

**Figure 1:**
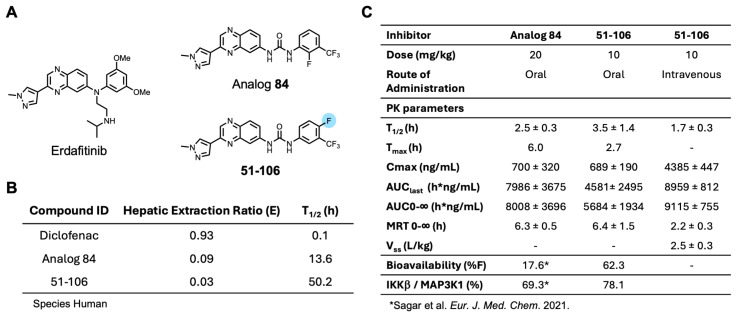
Development of **51**-**106** and PK studies. (**A**) Structures of analog **84** and **51**-**106**. The fluorine atom at the para-position of the phenyl ring in **51**-**106** is highlighted. This change was made to improve metabolic stability. (**B**) Diclofenac, analog **84**, and **51**-**106** subjected to human liver microsomes. The table lists the Hepatic Extraction Ratio and half-life for the compounds. (**C**) Oral and intravenous dose pharmacokinetic studies were performed in CD1 mice. The oral dose of **51**-**106** was 10 mg/kg, and the intravenous dose was 10 mg/kg. Data represented as average ± SD (*n* = 3) [25].

**Scheme 1:**
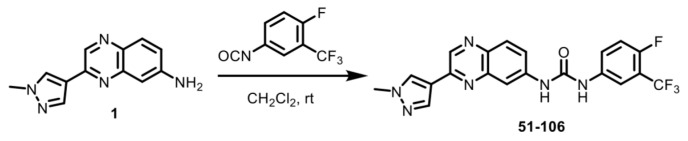
Synthesis of **51**-**106**. The regioisomer **51**-**106** was synthesized by coupling 3-(1-methyl-1H-pyrazol-4-yl)quinoxalin-6-amine (**1**) with freshly prepared 1-fluoro-4-isocyanato-2-(trifluoromethyl)benzene in dichloromethane.

An SAR study with a 55 member quinoxaline and quinoline analog library using a cell-based luciferase reporter assay, which specifically measures TNFα-induced IKKβ-mediated NFκB activation identified analog **84**, with an ortho F atom, meta -CF_3_ substituted phenyl quinoxaline urea, was the most potent inhibitor [25]. While analog **84** exhibited increased oral bioavailability (%F) over the starting compound **13**–**197**, it did not meet the industry minimum of ≥30% for a candidate to advance with manageable risk [26]. An approach to address this issue of bioavailability is to improve metabolic stability. The para-position of the phenyl ring in acetanilide moieties is susceptible to oxidation. We speculated that this might be the problem with **84**. To test this, we altered the substitution pattern on the phenyl ring by moving the F atom from the ortho in **84** to the para position (Figure 1A). The regioisomer **51**-**106**, resulting from the above change, was synthesized by coupling 3-(1-methyl-1H-pyrazol-4-yl)quinoxalin-6-amine (**1**) with freshly prepared 1-fluoro-4-isocyanato-2-(trifluoromethyl)benzene in dichloromethane (Scheme 1). To determine the metabolic stability of **51**-**106**, we incubated **51**-**106** and analog **84** with human (pooled male and female) liver microsomes and monitored their stability using liquid chromatography–tandem mass spectrometry. The FDA-approved drug Diclofenac was used as a positive control. The half-life (t_½_) of analog **84** was ~13 h, and **51**-**106** was ~50 h in human liver microsomes, an improvement in t_½_ of ~3.7-fold. The Hepatic Extraction Ratio (E) improved from 0.09 for analog **84** to 0.03 for **51**-**106** (Figure 1B). This suggests that the improved stability and bioavailability of **51**-**106** is associated with the reduced metabolic vulnerability due to the para-position blockade using the fluorine atom.

Next, we conducted pharmacokinetic (PK) studies with **51**-**106**. Briefly, mice were dosed either intravenously (IV) or orally with 10 mg/kg of **51**-**106** and sampled at 0.083, 0.25, 0.5, 1, 2, 4, 8, and 24 h. PK parameters summarized in Figure 1C show that t_1/2_ of **51**-**106** was 3.5 h, which is a ~1.4 fold improvement compared to that of analog **84**. Consistent with the increased microsomal stability, **51**-**106** exhibited a ~3.5-fold improvement of oral bioavailability from ∼17% for analog **84** [25] to ∼62% for **51**-**106** (Figure 1C). Taken together, our data suggest that the improvement in metabolic stability resulted in the improved bioavailability of **51**-**106**. This was accomplished by strategic placement of fluorine atoms at metabolically vulnerable sites on the molecule.

**Figure 2:**
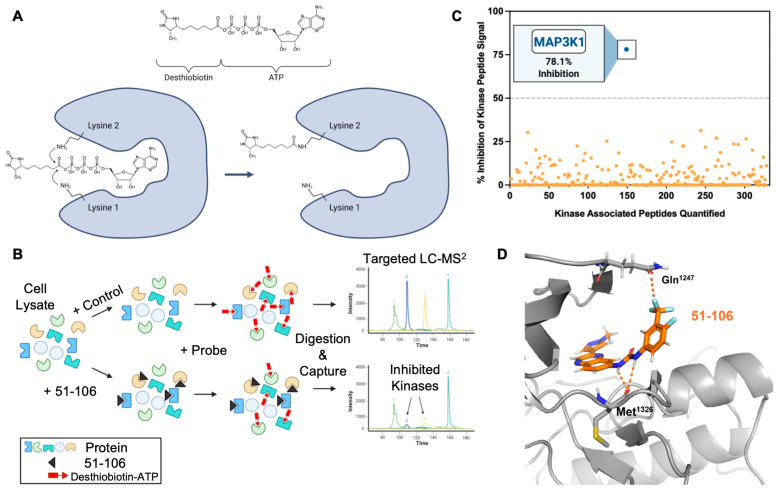
Chemoproteomic kinase profiling of **51**-**106**. (**A**) Chemical structure of desthiobiotinylated acylphosphate-nucleotide probe and schematic of the probe in ATP binding site of a kinase targeting a conserved lysine residue. (**B**) Schematic of the KiNativ method. Protein lysates are treated with inhibitor (**51**-**106**) followed by the addition of the acylphosphate probe. Proteins are digested by trypsin, and the biotinylated peptides are enriched by streptavidin. The probe-labeled peptides are quantified using a targeted LC-MS2 strategy. (**C**) Kinome profiling (KiNativ) of **51**-**106** in a pancreatic cancer cell line (PANC-1). Each colored dot represents a quantified kinase-associated peptide. This kinome dataset is the result of an analysis of duplicate treated and control samples. The % changes in MS signals being reported are statistically significant (*p*-value < 0.04, Student’s *t*-test). (**D**) There are no available structures of MAP3K1. We used the available AlphaFold MAP3K1 structure (AF-Q13233-F1-model.pdb) and docked **51**-**106** into the ATP binding site using Schrödinger GLIDE.

To assess target engagement and selectivity, we opted to conduct a kinome screen (KiNativ) in PANC-1 cell lysates [44,45]. This chemical proteomics (chemoproteomics) methodology allows for the evaluation of small-molecule binding to protein kinases in native systems such as cell lysates. KiNativ utilizes a soluble chemical probe comprising ATP linked to biotin through an acyl-phosphate bond. The probe covalently modifies conserved lysine residues found in the ATP binding site of essentially all protein kinases (Figure 2A). To profile a kinase inhibitor, cell lysates are treated with a vehicle control or inhibitor prior to addition of the desthiobiotin ATP probe. The ATP probe occupies the ATP binding site in the kinase, which enables the conserved lysines to be biotinylated. In kinases in which the inhibitor competes with the ATP probe, biotinylation does not occur, thus allowing the rapid identification of the target kinase and the selectivity for that kinase within the kinome in a single experiment (Figure 2B). The cell lysates are digested with a protease (trypsin), biotinylated peptides are affinity-enriched on streptavidin beads, and the labeled peptides are analyzed by liquid chromatography–tandem MS (Figure 2B). If an inhibitor binds to a kinase, it competes with the ATP, thus resulting in a loss of that kinase-associated peptide signal, allowing for the quantitative measurement of inhibitor binding. Unlike recombinant enzyme assays, KiNativ IC_50_ values are a direct measure of active site binding instead of enzymatic activity.

Kinome profiling quantified the activity of **51**-**106** against >300 kinases in the PANC-1 cells. The results summarized in Figure 2C are the average of duplicate control and duplicate treated samples with 5 μM of **51**-**106** or DMSO. Among 328 quantified kinases profiled, MAP3K1 exhibited 2.5-fold greater inhibition compared to any other kinase in the panel. The top five kinases inhibited besides MAP3K1 were PIK3CB (31.4%), CaMK1δ (30.3%), PKD3 (26.98%), STK33 (26.6%), and JAK3 domain2 (25.2%). Of the other MAP3Ks quantified, **51**-**106** did not inhibit MAP3K6 or MAP3K15 and inhibited MAP3K2, MAP3K3, MAP3K4, and MAP3K5 at a low level (4.6%, 11.4%, 8.6%, and 11.7%, respectively). Additionally, the MAP3K1 substrates IKKβ and MAP2K4 were not inhibited by **51**-**106**, and other members of MAPK signaling were also not substantially inhibited (Supplementary Figure S1). The results demonstrate that **51**-**106** selectively inhibits (78.1%) MAP3K1 (Figure 2C).

To determine the binding mode of **51**-**106**, we used Schrödinger GLIDE to dock **51**-**106** into the kinase domain of MAP3K1 (AlphaFold structure). The docked structure showed that the carbonyl oxygen atom of the MAP3K1 hinge region residue Met^1326^ is within hydrogen bonding distance (~3 Å) of the urea -NH atoms of **51**-**106**. We also noted that the -CF_3_ atom on the phenyl ring was close to the side chain carbonyl of Gln^1247^, potentially forming an orthogonal multipolar C-F····C=O with Gln^1247^ of MAP3K1, which has previously been shown to contribute to compound efficacy [46] (Figure 2D).

**Figure 3:**
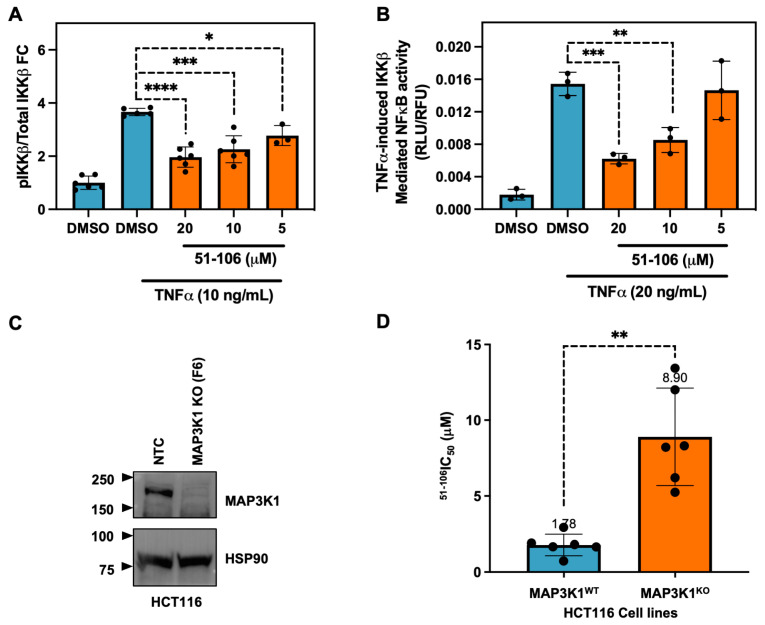
**51**-**106** inhibits MAP3K1 signaling, and its activity is MAP3K1 dependent. (**A**) Evaluation of TNFα induced MAP3K1 mediated phosphorylation of IKKβ using AlphaLISA assay. MiaPaCa2 cells (2 × 10^5^) were seeded in a 48-well plate and, after attachment, were serum-starved for 16 h. Cells were then treated with DMSO or **51**-**106** (20 μM, 10 μM, or 5 μM) for 30 min. The pathway was activated with 10 ng/mL TNFα and 10 nM Calyculin A for 10 min. AlphaLISA assay was conducted per manufacturer’s instruction for phospho-IKKβ and total IKKβ. Data represented as average ± SD with individual data points shown. *p*-value determined by two-tailed Student’s *t*-test, * *p* < 0.05, *** *p* < 0.001, **** *p* < 0.0001. (**B**) Evaluation of TNFα-induced NF-κB activation using luciferase reporter assay. Cervical cancer cells (HeLa) that stably express a luciferase reporter gene under the control of NF-κB response element were stimulated with TNF-α (20 ng/mL) for 6 h in the presence and absence of **51**-**106** (20 μM, 10 μM, or 5 μM). The NF-κB activity was measured using luciferase reagent at the indicated conditions and normalized to cell viability. Data represented as average ± SD with individual data points shown (*n* = 3). *p*-value determined by two-tailed Student’s *t*-test, ** *p* < 0.001, *** *p* < 0.0005. (**C**) Validation of MAP3K1 dependent effects. Colorectal cancer cell line (HCT116) MAP3K1 wild type (F10) and MAP3K1 KO (F6) cells (generated by the GES MSKCC) were subjected to Western blot and probed with anti-MAP3K1 and anti-HSP90 antibodies. (**D**) Growth inhibitory effects of **51**-**106** in HCT116 MAP3K1 wild type and MAP3K1 KO cells. Cells (4 × 10^3^/well) were subjected to increasing concentrations (0.02–20 μM) of **51**-**106** and incubated for 72 h. Cell growth was assessed using PrestoBlue reagent, and the data were fit to derive the IC_50_ values determined by curve-fitting the data (Prism version 9.1.0). *p*-value determined by two-tailed Student’s *t*-test with Welch’s correction, ** *p* < 0.005.

MAP3K1 was previously shown to phosphorylate IKKβ [12] and regulate NF-κB signaling [47]. Therefore, we validated the ability of **51**-**106** to inhibit MAP3K1 by probing the phosphorylation status of the known substrate, IKKβ, using an AlphaLISA assay. The pancreatic cancer cells (MiaPaCa2) were subjected to a 30-min pretreatment with varying concentrations of **51**-**106**, followed by TNFα stimulation to induce IKKβ phosphorylation. Following a 15-min stimulation, the levels of phospho-IKKβ and total IKKβ were quantified. The results from the AlphaLISA assay demonstrate that **51**-**106** inhibits TNFα-induced, MAP3K1-mediated IKKβ phosphorylation in a dose-dependent manner. (Figure 3A). Next, we evaluated the efficacy of **51**-**106** in a cell-based reporter assay utilizing a HeLa cell line that specifically responds to TNFα induced expression of a luciferase reporter gene that is under the control of an NF-κB response element. Consistent with the results from the AlphaLISA assay, we observed dose-dependent inhibition of TNF-α–induced MAP3K1-IKKβ–mediated NF-κB transcription by **51**-**106** (Figure 3B). These results suggest that **51**-**106** binds to MAP3K1 and blocks IKKβ phosphorylation, resulting in the inhibition of TNF-α-induced NF-κB activation.

To investigate the target dependency of **51**-**106** activity, MAP3K1 was genetically knocked out (KO) in HCT116 cells using CRISPR-Cas9 technology (Gene Editing and Screening Core Facility, Memorial Sloan Kettering Cancer Center). The HCT116 cell line was chosen because it is predominantly diploid, allowing for a more predictable outcome when utilizing CRISPR technology compared with cell lines with aneuploidy. Additionally, MAP3K1 has previously been successfully knocked out in HCT116 cells, suggesting the cells are viable [48]. Knockout efficiency was validated through TOPO cloning and CRISPR sequencing (Supplementary Figure S2), which revealed that 98% of MAP3K1 KO cells exhibited either a 5 base pair or 16 base pair deletion in exon 4 of MAP3K1, resulting in a frame-shift leading to premature stop codons. Western blot analysis confirmed the absence of MAP3K1 protein expression in KO cells compared to non-targeting control (NTC) MAP3K1 wild-type (WT) cells (Figure 3C). The MAP3K1 primary antibody used binds to residues 50 and 100 of the N-terminus of MAP3K1. HCT116 MAP3K1 WT and MAP3K1 KO cells were subjected to PrestoBlue growth inhibition assay with **51**-**106** treatment. **51**-**106** had an IC_50_ of ~1.8 μM in MAP3K1 WT cells and ~8.9 μM in MAP3K1 KO cells (Figure 3D). The 5-fold increase in IC_50_ in MAP3K1 KO cells suggests that the efficacy of **51**-**106** is dependent on the presence of MAP3K1, further validating MAP3K1 as the primary target of **51**-**106**. While **51**-**106** perturbs MAP3K1, it does not meet the cutoff of at least 30-fold selectivity of the ligand towards the target protein over competing proteins [49]. Further optimization of **51**-**106** and/or the use of emerging methods such as DNA-encoded library based screens followed by machine learning (DEL-ML) along with a comprehensive suite of biophysical and biochemical screens should be employed to develop a MAP3K1 chemical probe [50,51].

**Figure 4:**
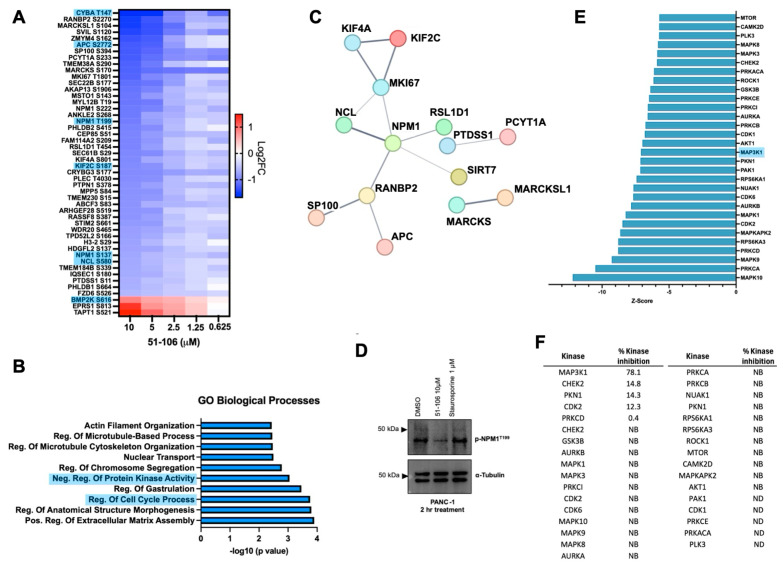
Phosphoproteomic analysis following MAP3K1 inhibition with **51**-**106** treatment. PANC-1 cells were treated with either DMSO or **51**-**106** (10 µM, 5 µM, 2.5 µM, 1.25 µM, 0.625 µM) for 2 h. The were processed and analyzed for phosphoproteomic and total proteomic analysis using LC-MS/MS analysis. (**A**) Heatmap of the significant sites identified with RokaiXplorer analysis, which decreased (blue) or increased (red) in a **51**-**106** dose-dependent manner (log2 FC > ±0.32, FDR < 0.05) across the 10 µM, 5 µM, 2.5 µM, and 1.25 µM doses. Proteins known to interact with MAP3K1 from the Pathway Commons Database are highlighted in blue. (**B**) Top 10 significant GO Biological Processes enriched using Enrichr program, containing Ser/Thr-Pro phosphosites that decrease in a dose-response fashion with **51**-**106** treatment. (**C**) STRING network of medium-confidence interactions (minimum confidence score of 0.400) among proteins containing Ser/Thr-Pro phosphosites that decrease in a dose-dependent manner with **51**-**106** treatment. Disconnected nodes are not shown. Color of the nodes is arbitrary. (**D**) Western blot validation of phosphoproteomics results of NPM1 T199 phosphorylation inhibition. PANC-1 cells were treated with DMSO, **51**-**106**, or Staurosporine, a multi-kinase inhibitor, for 2 h and membrane probed for phosphorylation NPM1 at T199 and α-Tubulin loading control. (**E**) The top 30 predicted inhibited kinases based on substrate phosphorylation changes by RokaiXplorer (FDR < 0.05) sorted by Z-score. (**F**) Results from the KiNativ kinome screen of the top 30 predicted kinases. NB = no binding, ND = not detected.

Next, we wanted to assess the effects of **51**-**106** on the early phosphorylation events associated with transient MAP3K1 inhibition. To this end, we conducted global proteomic and phosphoproteomic analyses of PANC-1 cells treated with varying doses of **51**-**106** (10, 5, 2.5, 1.25, 0.625, and 0 μM) for 2 h using a tandem mass tag (TMT)-based multiplexing strategy (Figure 4). Across all conditions, a total of 7028 proteins and 22,843 phosphosites were quantified and successfully mapped to known protein sequences. Significance cutoffs of >0.32 or <−0.32 log_2_FC of **51**-**106** compared to DMSO and FDR < 0.05 were used to analyze differences in total and phosphorylated protein levels between treatment and control samples using RokaiXplorer (Supplementary Figure S3a) [52,53]. The total protein levels were not altered significantly, as the incubation time was relatively short (2 h) (Supplementary Figure S3b). Of the 445 phosphosites that were significantly altered across the 10, 5, 2.5, and 1.25 μM doses, the levels of 329 phosphosites were decreased, and 116 phosphosites were increased.

We observed that the abundance of forty-five phosphosites was decreased, and three phosphosites were increased in a dose-dependent manner. Among these proteins, there were several known MAP3K1 interactors (Pathway Commons Database) (Figure 4A) [54]. This is consistent with our finding that **51**-**106** is a selective MAP3K1 inhibitor. The protein that had the greatest phosphorylation fold change at any dose was NPM1, with a 3.5-fold decrease in S227 observed after 10 μM treatment with **51**-**106**. Additionally, NPM1 had multiple phosphorylation sites that were decreased in a dose-dependent fashion across all doses. Phosphorylation changes in MAPK signaling pathway proteins were also investigated, and several known MAP3K1 pathway members were shown to decrease with **51**-**106** treatment, including ERK1/2, MAPK14, and NFκB2 (Supplementary Figure S3c). Significant changes in proteins known to interact with MAP3K1 from the Pathway Commons database are summarized in Supplementary Figure S3d. Due to the nature of phosphoproteomics profiling, some known substrates of MAP3K1 were not quantified in this dataset because of low abundance, including S177 of IKKβ. This is not uncommon, as we were unable to detect this region of IKKβ even when recombinant IKKβ was subjected to proteomic analysis [55].

Gene ontology analysis using the open-source platform Enrichr [56,57,58] was used to investigate the biological processes associated with the proteins that had phosphosites that decreased in a dose-dependent manner. The top enriched pathways associated with these downregulated proteins included regulation of protein kinase activity, regulation of cell cycle processes, regulation of microtubule cytoskeleton organization, and actin filament organization (Figure 4B). Proteins with phosphosites that decreased in a dose-dependent manner were examined for interactions using the STRING database [59,60], which identified NPM1 as the most interconnected protein (Figure 4C).

We further investigated changes in NPM1 phosphorylation in our dataset. Of the NPM1 phosphorylation sites quantified, four sites had significant dose-dependent decreases upon **51**-**106** treatment: S137, T199, S222, and S227. Only one of these phosphorylation sites, T199, has a commercially available antibody. Importantly, T199 phosphorylation plays an essential role in NPM1 function, as it is required for localization of NPM1 to sites of DNA double-stranded breaks, where it colocalizes with other DNA repair proteins such as γH2AX and RAD51 [38]. Decreased phosphorylation in NPM1 T199 was validated via Western blot analysis of PANC-1 cells treated with **51**-**106** or Staurosporine (non-selective kinase inhibitor) for 2 h (Figure 4D).

In addition to estimating fold changes at a specific phosphorylation site between treatment and control, the activity of the upstream kinases was predicted using RokaiXplorer based on these changes in phosphosites. The functional network used by the algorithm to infer kinase activity includes kinase–substrate associations, protein–protein interactions between kinases, and structure distance and co-evolution evidence for interactions between phosphosites [52,53]. The top 30 kinases predicted to have decreased activity based on **51**-**106** treatment included several MAP kinases, including MAPK8, MAPK3, and MAP3K1 (Figure 4E). Of those top 30 kinases, the activity of **51**-**106** against 25 were quantified in the KiNativ kinome screen. Among the twenty-five quantified, twenty-one of the kinases exhibited no binding to **51**-**106**, three kinases exhibited less than 15% inhibition, and only MAP3K1 was inhibited by 78% (Figure 4F). NPM1 is known to be phosphorylated by CDK2/Cyclin E [31,61], and the KiNativ data show that CDK2 was one of the three kinases that was inhibited by 12% by **51**-**106** (Figure 4F). While we cannot completely rule out the contribution of CDK2 inhibition resulting in reduced T199 phosphorylation, our data strongly suggest that under basal conditions in pancreatic cancer cells, NPM1 T199 phosphorylation is mediated by MAP3K1. This is consistent with reported studies that show NPM1 to be a member of the NF-κB signaling axis [62], and MAP3K1 and NPM1 physically interact [63]. However, this is the first study to show a link between inhibition of MAP3K1 kinase function and NPM1 phosphorylation. Further investigation into MAP3K1-NPM1 signaling is needed to determine if MAP3K1 directly phosphorylates NPM1 at T199, or if MAP3K1 phosphorylates a different kinase upstream of NPM1.

## 3. Materials and Methods

^1^H NMR and ^13^C NMR spectra were measured on a 500 MHz spectrometer (Bruker, Billerica, MA, USA, TopSpin 3.6.5), using CDCl_3_ and DMSO-*d*_6_ as the solvents with tetramethyl silane (TMS) as an internal standard at room temperature. High-resolution mass spectra (HR-MS) were acquired using an Agilent 6230 LC/TOF mass spectrometer (Agilent technology Co., Ltd., Santa Clara, CA, USA). All solvents used in the experiment were dried using activated molecular sieves, and the other reagents used in the experiment were all analytically pure without any other treatment. Chemical shifts are given in δ relative to TMS, and the coupling constants *J* are given in Hz.

### 3.1. Synthesis of 1-(4-Fluoro-3-(trifluoromethyl)phenyl)-3-(3-(1-methyl-1H-pyrazol-4-yl)quinoxalin-6-yl)urea *(**51**-**106**)*

3-(1-methyl-1H-pyrazol-4-yl)quinoxalin-6-amine (**1**, 50 mg, 0.22 mmol) was synthesized following methods previously reported by us [25]. Dichloromethane was placed in a round bottom flask. To this solution was added freshly prepared 1-fluoro-4-isocyanato-2-(trifluoromethyl)benzene (Matrix fine chemicals, 68.3 mg, 0.33 mmol). The mixture was stirred at 50 °C for 12 h. After competition of the reaction, the precipitates formed were filtered and redissolved in a mixture of ethyl acetate and MeOH (98:2). The solution was loaded on a silica column and purified using ethyl acetate/MeOH as an eluting solvent; yield = 81%; ^1^H NMR (400 MHz, DMSO) δ 9.35 (s, 1H), 9.24 (s, 1H), 9.13 (s, 1H), 8.62 (s, 1H), 8.27 (s, 1H), 8.25 (d, *J* = 2.3 Hz, 1H), 8.06 (dd, *J* = 6.3, 2.6 Hz, 1H), 7.95 (d, *J* = 9.0 Hz, 1H), 7.71 (dd, *J* = 8.9, 2.5 Hz, 2H), 7.49 (t, *J* = 9.8 Hz, 1H), 3.96 (s, 3H); ^13^C NMR (100 MHz, DMSO) δ 155.5, 153.0, 147.8, 143.1, 142.0, 141.4, 138.4, 137.2, 136.7, 131.4, 129.7, 125.0, 124.9, 124.4, 122.3, 120.7, 118.2, 118.0, 116.8, 116.7, 114.1; HRMS (ESI-MS) calcd for C_20_H_15_F_4_N_6_O^+^ *m*/*z* (M + H)^+^ 431.1238, found: 431.1380.

### 3.2. Metabolic Stability and Pharmacokinetics Studies

The in vitro metabolic stability of **51**-**106** was determined using human liver S9 fraction (XenoTech, LLC; Kansas City, KS, USA), following previously reported methods [64]. Diclofenac (CYP hydroxylation) was used as a positive control for the in vitro metabolic stability study. Sample analysis was performed by liquid chromatography–tandem mass spectrometry (SCIEX QTRAP 4000 LC-MS/MS System, SCIEX, Framingham, MA, USA).

Pharmacokinetic studies (10 mg/kg oral and intravenous dosing) were performed following previously reported methods [64]. Sample analysis was performed by liquid chromatography–tandem mass spectrometry (SCIEX QTRAP 4000 LC-MS/MS System). The pharmacokinetic parameters were determined using non-compartmental analyses module of WinNonlin 1.5.

### 3.3. AlphaLISA Reporter Assay

A suspension of MiaPaCa2 cells (2 × 10^5^/well) in 200 μL media was plated in a flat, clear-bottomed, sterile, 48-well tissue culture plate (Cat# 353230; Corning; Corning, NY, USA). The cells were allowed to attach to the plate for ~8 h in a humidified 5% CO_2_ incubator at 37 °C. The media was then removed, replaced with serum-free media, and incubated for 16 h in a humidified 5% CO_2_ incubator at 37 °C. The cells were treated with DMSO control or with **51**-**106** 20 μM, 10 μM, or 5 μM concentration and incubated for 30 min in a 5% CO_2_ incubator at 37 °C. Cells were then treated with 10 ng/mL TNFα (Cat# 300-01B-20UG; Thermo Fisher Scientific; Waltham, MA, USA) (and 10 nM Calyculin A (Cat# PHZ1044; Thermo Fisher Scientific) for 10 min. Media was aspirated, cells were lysed using 50 μL of lysis buffer from the AlphaLISA SureFire Ultra Human Phospho-IKKβ (Cat# ALSU-PIKKB-A500; Revvity; Waltham, MA, USA) or Total IKKβ (Cat# ALSU-TIKKB-A500, Revvity) kit, and the plate was shaken at RT for 45 min. Using 20 μL of lysate for a final reaction volume of 40 μL, the assay was conducted per manufacturer’s instructions, and the plate was read on a Spectramax i3X using standard AlphaLISA settings (Molecular Devices, San Jose, CA, USA). Phospho-IKKβ levels were normalized to total phospho-IKKβ, and fold change was calculated compared to DMSO treatment. Data were plotted using Prism V 9.1.0. The statistical significance between groups was determined via unpaired *t*-tests coupled with post hoc analysis with Welch’s correction.

### 3.4. Luciferase Reporter Assay

A suspension of HeLa-NFkB-Luc cells (Cat# SL-0001; Signosis; Santa Clara, CA, USA) (2 × 10^4^/well) in 80 μL media was plated in white-walled, flat, clear-bottomed, sterile, 96-well tissue culture plates (Cat# 3610; Corning). The cells were allowed to adhere overnight in a humidified 5% CO_2_ incubator at 37 °C. Drug working solutions were prepared from 10 mM DMSO stocks. Drugs were added in 3 dilutions in triplicates. 10 μL of the working solution was added to each well in the plate to yield the indicated concentration of (20—0.6 μM) drug in 0.1% DMSO. Blank DMSO controls were also included. The cells were incubated, after the addition of the drugs, for 15 min at 37 °C. The cells were stimulated with 10 μL of TNFα (Cat#300-01A-100UG; Thermo Fisher Scientific), except DMSO controls. The cells were returned to the incubator and further incubated for 3 h. Ten μL AlamarBlue (Cat# BUF012B; Bio-Rad; Hercules, CA, USA) was added to all wells, and the plates were returned to the incubator for another 3 h. Post stimulation addition (6 h), fluorescence (Ex 560, Em 590) was measured on the plate reader to assess cell viability. The plates were then allowed to equilibrate and attain room temperature. A total of 110 μL of ONE-Glo luciferase reagent (Cat# E6120; Promega; Madison, WI, USA) was added per well, and the plates were shaken well. Luminescence (1000 ms) was measured on the plate reader set to the kinetic mode of acquisition for 15 min. The luminescence data were normalized to the AlamarBlue values. Changes were reported as compared against a DMSO control for the 8 min time point.

### 3.5. Cell Viability Assay

A suspension of in 90 μL media was plated per well in clear, flat-bottomed, sterile, 96-well tissue culture plates (Cat# 12-565-438; Fisher Scientific, Waltham, MA, USA). The cells were allowed to adhere overnight in a humidified 5% CO_2_ incubator at 37 °C. Drug working solutions were prepared from 10 mM DMSO (Cat# BP231; Fisher Scientific) stocks. Drugs were added in 6 dilutions in triplicates. Ten μL of the working solution was added to each well in the plate to yield a final concentration of 20—0.63 μM drug in 1% DMSO. Blank DMSO controls were also included. Once drugs were added, 10 μL Presto blue/well (Cat# A13262; Invitrogen; Waltham, MA, USA) was added to three wells containing 1% DMSO and incubated at 37 °C for 15 min. Fluorescence (Ex 560, Em 590) was measured on a plate reader (Spectramax M5e; Molecular Devices) to determine T0. The plates were returned to the incubator and further incubated for 72 h. After 72 h, 10 μL Presto blue/well was added to the remaining wells and incubated at 37 °C for 15 min. Fluorescence was measured on a plate reader. Wells with 1% DMSO were used to determine T100. % Growth was calculated as [(Fluorescence read of a well − T0)/(T100 − T0)] ∗ 100. The data were plotted using Prism V 9.1.0, and the IC_50_ values were determined using a four-parameter Logistic Curve Fitting function.

### 3.6. Western Blotting

Cells were washed with cold 1× PBS and lysed in Radioimmunoprecipitation assay (RIPA) buffer (Cat# 89901; Thermo Fisher Scientific), sodium orthovanadate (Na_3_VO_4_) (Cat# 567540-5GM; Sigma-Aldrich; St. Louis, MO, USA), sodium fluoride (NaF) (Cat# 201154-5G, >99% purity; Sigma-Aldrich), β-glycerophosphate (Cat# G9422-10G; Sigma-Aldrich), and 1 mM phenylmethylsulfonyl fluoride (Cat# 10837091001; Sigma Aldrich). The samples were incubated on ice for 30 min, vortexed at 10 min intervals, and centrifuged at 14,000× *g* for 10 min at 4 °C. The supernatant was collected, and protein was quantified using the BCA Protein Assay kit (Cat# 23225; Thermo Fisher Scientific). Proteins were separated in 4–15% polyacrylamide gradient gel (Cat# 4561086; Bio-Rad) in 1×TRIS-Glycine-SDS Buffer (Cat# T32080; Research Products International Corporation; Mount Prospect, IL, USA) at 120 V for ~80 min. The proteins were transferred to a polyvinylidene fluoride (PVDF) membrane (Cat# IPVH00005; Sigma-Aldrich) by semi-dry transfer method (Cat# 35035; Thermo Fisher Scientific) at 18 V for ~35 min. The membranes were blocked by adding 5% (*w*/*v*) non-fat dry milk (5% BSA for phospho-antibodies) in 1×-Tris-Buffered Saline with 0.1% Tween (1× TBST) (Cat# J60764.K2; Thermo Fisher Scientific) (Cat# P9416; Sigma-Aldrich) for 1 h at room temperature (RT). This was followed by incubation with the primary antibodies diluted in 5% milk in 1× TBST at 4 °C overnight with gentle rocking. The primary antibodies used were MAP3K1 (Cat#A302-395A; Bethyl Laboratories; Montgomery, TX, USA), HSP90 (Cat# 4877; Cell Signaling Technology; Danvers, MA, USA). After washes with 1× TBST (3 times for 10 min each), the membranes were incubated with the appropriate HRP-conjugated secondary antibody (Cat# 31460; Thermo Fisher Scientific) for 1 h at RT with gentle rocking. The membranes were washed again (3 times for 10 min each) with 1× TBST. The membranes were then incubated with ECL Select (Cat# RPN2235; Cytiva; Marlborough, MA, USA) to detect protein levels.

### 3.7. Proteomics

PANC-1 (1 × 10^7^) cells were seeded into T125 flasks (FB012939, Fisher Scientific) and allowed to attach overnight in a humidified 5% CO_2_ incubator at 37 °C. Drug working solutions were prepared from 10 mM DMSO stocks. **51**-**106** was added in 4 dilutions, in triplicates. 20 μL of the working solution was added to each plate to yield the indicated concentration of (10—0.625 μM) drug in 0.1% DMSO. Plates were incubated in a humidified 5% CO_2_ incubator at 37 °C for 2 h. Media was removed from the plate by aspiration and washed with ice-cold PBS before incubation with trypsin for 5 min. Cells were collected in media by centrifugation at 2000 rpm for 5 min at RT and washed with ice-cold PBS twice. Cell pellets were collected by centrifugation at 17,000× *g* for 20 min at 4 °C and stored at −80 °C until shipment.

In brief, the experimental strategy for global proteomics and phosphoproteomics is as follows: The cell pellets were subjected to protein extraction in 8 M urea/50 mM TEABC pH 8.5 lysis buffer. Following reduction in 5 mM dithiothreitol and alkylation in 15 mM iodoacetamide, proteins were subjected to trypsin digestion in 1:50 enzyme-to-protein ratio overnight at 37 °C. Tryptic peptides were purified using Sep-Pak C_18_ cartridges and then labeled with TMT 18-plex reagents per the manufacturer’s instructions. Peptides were then pooled and fractionated into 12 fractions using basic pH reversed-phase HPLC fractionation system. A total of 5% of each fraction was used for the global proteomic analysis, and the remaining 95% of the fraction was subjected to phosphopeptide enrichment using Fe(III)-NTA IMAC cartridges on Bravo automated liquid handling system (Agilent). Fractions for total proteomic and Phosphoproteomic analysis were dried and resuspended in 0.1% formic acid for LC-MS/MS analysis.

Global proteome and phosphopeptide-enriched fractions were analyzed on an Orbitrap Eclipse mass spectrometer using UltiMate 3000 RSLC nano system for online separation of peptides in trap and elute configuration (Thermo Scientific, San Jose, CA, USA). Peptides were initially trapped onto a trap column (Optimize Technologies, Oregon City, OR, USA) using solvent A (0.1% formic acid) and then separated on an analytical column (PepSep 50 cm × 75 μm, C_18_ 1.9 μm) using a gradient of solvent B (Acetonitrile, 0.1% formic acid) from 6% to 32% at 0.3 µL/min flow rate. The gradient was followed by a high organic wash to clean the column for the next injection. Analytical column was heated at 55 °C using butterfly portfolio heater (Phoenix S&T, Chadds Ford, PA, USA) to maintain the back pressure. Total time for each run was 155 min. Mass spectrometry analysis was performed in a data-dependent mode with a cycle time of 2 s. Survey MS scans were recorded in Orbitrap mass analyzer with 375 to 1500 m/z scan range at 120,000 resolution. Each MS scan was acquired with 400,000 AGC target and a maximum injection time of 50 ms. MS/MS events were sequentially triggered based on the abundance of the precursor ions with a minimum intensity threshold of 5000 and charge states 2–7. Precursors ions were filtered by quadrupole using an isolation width of 0.7 *m*/*z* for global proteome and 1.2 *m*/*z* for phosphopeptide-enriched fractions and subjected to fragmentation using high-energy collision-induced dissociation (HCD) at 34% normalized collision energy. Fragment ion scans were acquired in Orbitrap mass analyzer at 30,000 resolution, AGC target of 100,000, and maximum injection time of 100 ms. Enhanced resolution mode for TMTpro reagents was activated for the MS/MS analysis. Dynamic exclusion setting of 40 s was used to prevent repeated triggering of MS/MS events.

Raw data were analyzed using Proteome Discoverer 2.5 (Thermo Scientific). Database searching was performed against UniProt human protein database along with contaminant proteins. In silico protein digestion was performed using trypsin with full cleavage specificity, maximum missed cleavages of 2, minimum and maximum peptide length of 7 and 50. Precursor and fragment ion tolerance of 10 ppm and 0.05 Da was used for spectral matching. Oxidation at methionine as dynamic modification, TMT modification at peptide N-termini, lysine and carbamidomethylation at cysteine were specified as static modifications. Additionally, for phosphoproteomic analysis, phosphorylation at serine, threonine, and tyrosine were specified as dynamic modification. Percolator node was used to control the false discovery rate at 1% protein and peptide levels. Reporter ion quantitation was performed using most confident centroid method with an integration tolerance of 20 ppm. Peptide spectral matches with the average reporter S/N threshold below 20 were filtered out for quantitation. IMP-ptmRS node was used for phosphoproteomic analysis to calculate the site probability for phosphosites. Normalization was performed using total peptide amount mode within Proteome Discoverer software.

Fold changes were calculated for each dose in comparison with DMSO. RokaiXplorer (https://rokai.io/explorer/, accessed on 16 July 2024) was used to analyze the changes in treatment compared to DMSO, with significance cutoffs of log2 FC < −0.32 or log2 FC > 0.32, *p*-value < 0.05, FDR < 0.05. RokaiXplorer was used to conduct upstream kinase analysis with a minimum number of 4 substrates using the PhosphoSitePlus and Signor databases and the *KS + PPI + SD + COEv* RoKAI functional network, *p*-value < 0.05, FDR < 0.05. Data plots were generated using Prism V 9.1.0. The STRING database (https://string-db.org, accessed on 16 July 2024) was used to generate a network of medium-confidence interactions (minimum confidence score of 0.400) among proteins containing phosphosites that decreased in a dose-response fashion to **51**-**106** treatment with disconnected nodes removed. Enrichr (https://maayanlab.cloud/Enrichr/, accessed on 16 July 2025) was used to search for enriched terms in the GO: Biological Processes database.

### 3.8. Docking Studies

The AlphaFold model was downloaded (https://alphafold.ebi.ac.uk/entry/Q13233, accessed on 30 September 2023). Molecular docking was conducted using the Maestro Version 11.5.011 (Release 2018-1) from the Schrödinger Suite. MAP3K1 AlphaFold structure (AF-Q13233-F1-model.pdb) was subjected to protein preparation wizard in the default mode. Ligprep (OPLS-2005 force field) from the Schrodinger suite was used to prepare **51**-**106**. All possible protonation states were enumerated for the ligand using Epik at a pH of 7.0. Tautomeric states were generated for chemical groups with possible tautomerism. Receptor grid box was generated by specified residue (Met1326) in the active and ligand binding site of the protein using the OPLS-2005 force field. The optimized ligand was then docked with Schrödinger’s Glide software (Release 2018-1).

## 4. Conclusions

In summary, we report the discovery of a selective MAP3K1 inhibitor, **51**-**106**, with excellent pharmacokinetic properties. Using orthogonal assays systems, we demonstrate that **51**-**106** perturbs the MAP3K1-IKKβ-NFκB pathway. The value of **51**-**106** as a small molecule MAP3K1 inhibitor was demonstrated using a phosphoproteomic study that led to the identification of NPM1 as member of the MAP3K1 network. Studies focused on exploring **51**-**106** as a therapeutic option for pancreatic cancer are currently underway and will be reported in due course.

## Data Availability

Data will be made available from the authors upon reasonable request. The mass spectrometry data have been deposited to the Proteome Xchange Consortium via the PRIDE partner repository with the dataset identifier PXD055363.

## Supplementary Materials

The following supporting information can be downloaded at https://www.mdpi.com/article/10.3390/molecules30092001/s1: Figure S1: Kinome profiling (KiNativ) of **51**-**106** among MAPK pathway kinases; Figure S2: Validation of MAP3K1 KO in HCT116 cells (GES, MSKCC); Figure S3: Phoshoproteomic analysis following **51**-**106** treatment.
