# Supplementary material for: A Selective MAP3K1 Inhibitor Facilitates Discovery of NPM1 as a Member of the Network
Source: Molecules. 2025 Apr 30;30(9):2001. doi: 10.3390/molecules30092001 (PMC12073402; doi:10.3390/molecules30092001)

Supplementary Materials

A selective MAP3K1 inhibitor facilitates discovery of NPM1 as a member of the network

Lidia Boghean [1], Sarbjit Singh [1], Kiran K. Mangalparthi [2], Smitha Kizhake [1], Lelisse Umeta [1], Donn Wishka [3], Paul Grothaus [3], Akhilesh Pandey [2,4], and Amarnath Natarajan [1, 5*]

[1]Eppley Institute for Research in Cancer and Allied Diseases, University of Nebraska Medical Center, Omaha, NE, USA
[2]Department of Laboratory Medicine and Pathology, Center for Individualized Medicine, Mayo Clinic, Rochester, MN, USA
[2]Drug Synthesis and Chemistry Branch, National Cancer Institute, NIH, Bethesda, MD, USA
[4]Manipal Academy of Higher Education, Manipal, Karnataka, India
[5]Fred and Pamela Buffett Cancer Center, University of Nebraska Medical Center, Omaha, NE 68198, USA
*Author to whom correspondence should be addressed. anatarajan@unmc.edu ; Tel.: +1-(402)-559-3795

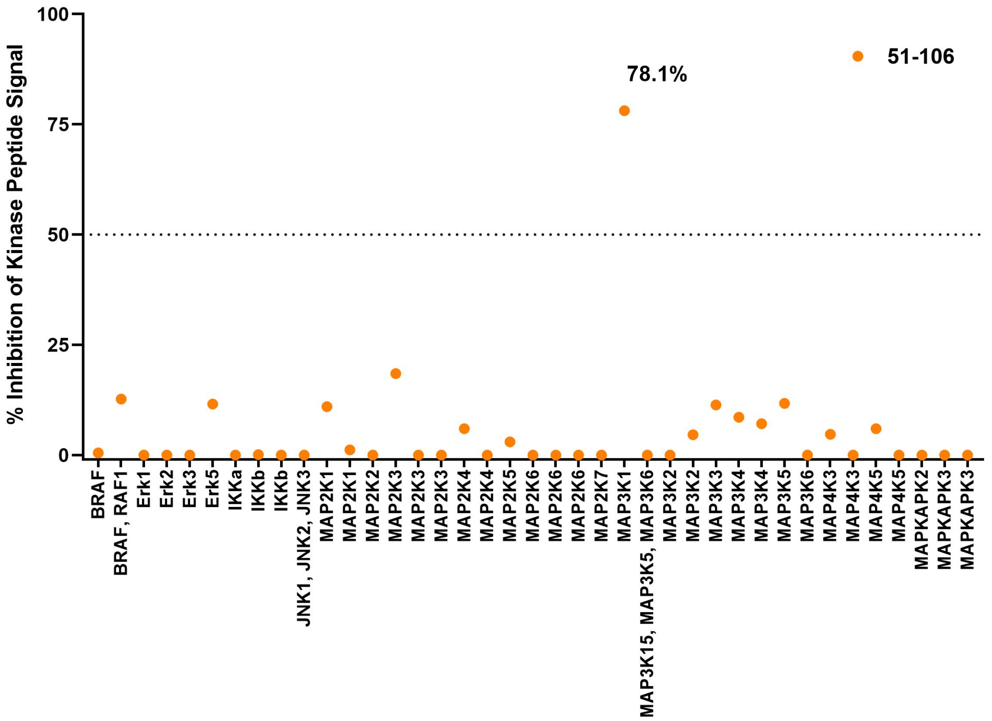

Supplementary Figure S1. Kinome profiling (KiNativ) of 51-106 among MAPK pathway kinases. Each colored dot represents a quantified kinase associated peptide. This kinome dataset is the result of an analysis of duplicate treated and control samples. The % changes in MS signals being reported are statistically significant (p-value < 0.04, Student's t-test).

a

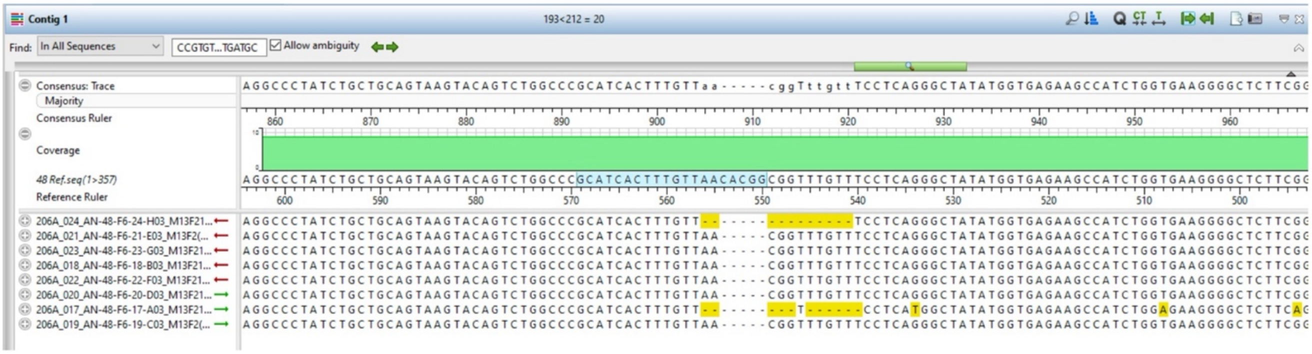

b

	Target Reads	Mutant Reads	% Mutant	Genotype	Frame Shift Mutant Reads	% Frameshift Mutant	% 5 bp deletion	% 16 bp deletion
HCT 116 MAP3K1 KO (F6)	279043	274842	98.49	Homozygous Mutant	269350	96.53	46.95	41.71

Supplementary Figure S2. Validation of MAP3K1 KO in HCT116 cells (GES, MSKCC). **(A)** TOPO Cloning data shows a 5 bp and 16 bp deletion in the HCT116 MAP3K1 KO clone F6. **(B)** CRISPR sequencing data for HCT116 MAP3K1 KO clone F6 shows 98.49% homozygous mutations resulting in 96.53% frameshift mutations. The majority of the reads exhibited 16 bp deletions (46.95%) and 5 bp deletions (41.71%) and only 1.33% of reads were WT. Both 16 bp and 5 bp deletions cause the creation of multiple stop codons in Exon 4 of MAP3K1.

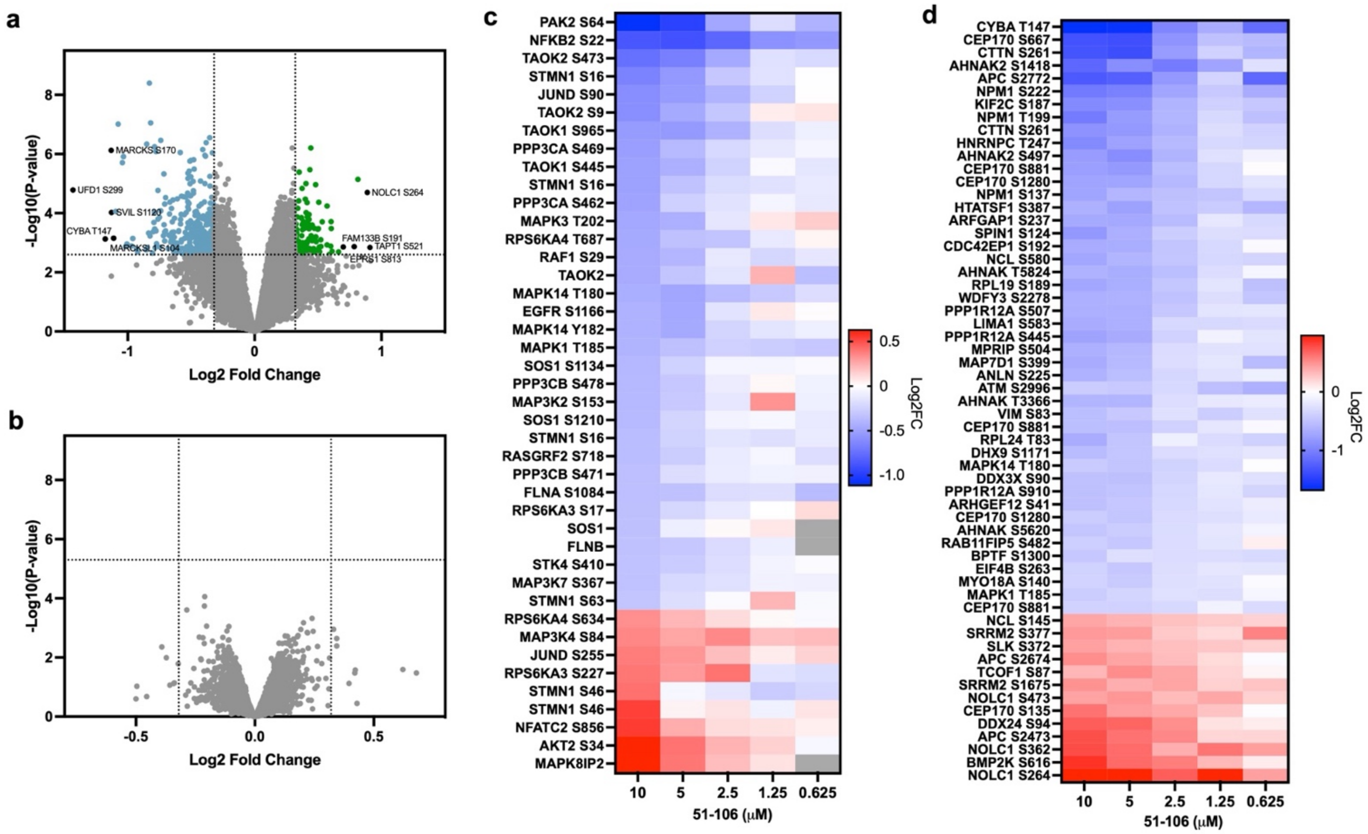

Supplementary Figure S3. Phoshoproteomic analysis following 51-106 treatment. PANC-1 cells were treated with either DMSO or a dose response of 51-106 (10 μM, 5 μM, 2.5 μM, 1.25 μM, 0.625 μM) for 2 hr before cell pellets were collected and analyzed for phosphoproteomic and total proteomic analysis. Data was analyzed using RokaiXplorer. **(a)** Volcano plot of phosphorylation sites quantified plotted by Log2 Fold Change (average 10 μM, 5 μM, 2.5 μM, 1.25 μM treatment vs. DMSO) and –log10(P-value). Significantly increased phosphorylation sites are colored in green (log2 FC >0.32, FDR < 0.05) and significantly decreased phosphorylation sites are colored in blue (log2 FC <-0.32, FDR < 0.05). Top 5 significantly increased and top 5 significantly decreased phosphorylation sites are colored in black and labeled with their corresponding gene name and phosphorylation site. **(b)** Volcano plot of total protein changes in the dataset. No proteins significantly changed (log2 FC > 0.32 or log2 FC < -0.32, FDR < 0.05). **(c)** Heatmap of phosphosites of proteins in the KEGG MAPK pathway that showed log2 FC < -0.32 or log FC > 0.32 at the highest dose of 51-106 treatment. **(d)** Heatmap of phosphosites of known MAP3K1 interactors from the database that showed log2 FC < -0.32 or log FC > 0.32 at the highest dose of 51-106 treatment and FDR <0.05

KiNativ assay with 51-106.

Labeling Site Key

**Lys1:**	Conserved Lysine 1
**Lys2:**	Conserved Lysine 2
**ATP Loop:**	ATP binding loop
**Activation Loop:**	Activation loop
**ATP:**	ATP site in non-canonical kinase (e.g. lipid kinase)
**Protein Kinase Domain:**	Other lysine within kinase domain, possibly not in ATP binding site
**Other:**	Labeling of residue outside of the protein kinase domain, possibly not in ATP binding site

	>90% Inhibition
	75 - 90% Inhibition
	50 - 75% Inhibition
	35 - 50% Inhibition
	No change. NB = No Binding (≤ 0)
	>100% increase in MS signal (>2 fold increase)
ND	Not determined

Data points inhibited >35% & not considered significant are left uncolored

Note: This KiNativ dataset is the result of an analysis of duplicate treated samples and control samples.
The % changes in MS signals being reported are statistically significant (Student T-test score < 0.04)

Chemoproteomic profiling in PANC1 lysate

Kinase	Reference	Sequence	Labeling Site	51-106 5 µM
AAK1	Q2M2I8	DLKVENILLHDR	Lys2	3.5
ABL, ARG	P00519, P42684	YSLTVAVKTLK	Lys1	3.5
ABL, ARG	P00519, P42684	LMTGDTYTAHAGAKFPIK	Activation Loop	NB
ABL, ARG	P00519, P42684	YSLTVAVKTLKEDTMEVEEFLK	Lys1	NB
ACK	Q07912	TVSVAVKCLKPDVLSQPEAMDDFIR	Lys1	NB
ACK	Q07912	KVPFAWCAPESLK	Activation Loop	NB
AGK	Q53H12	ATVFLNPAACKGK	ATP	NB
AKT1	P31749	GTFGKVILVK	ATP Loop	NB
AMPKa1, AMPKa2	Q96E92, P54646	DLKPENVLLDAHMNAK	Lys2	8.7
AMPKa1, AMPKa2	Q96E92, P54646	VAVKILNR	Lys1	7.5
ARAF	P10398	DLKSNNIFLHEGLTVK	Lys2	NB
ATM	Q13315	QLVKGRDDLRQDAVMQQVFQMCNTLLQR	ATP	7.4
ATR	Q13535	FYIMMCKPK	ATP	2.2
AurA	O14965	FILALKVLFK	Lys1	NB
AurA	O14965	DIKPENLLLGSAGELK	Lys2	NB
AurA, AurB, AurC	Q9UQB9, Q96GD4, O14965	GKFGNVYLAR	ATP Loop	NB
AurB	Q96GD4	SHFIVALKVLFK	Lys1	NB
AXL	P30530	KIYNGDYYR	Activation Loop	NB
BARK1	P25098	DLKPANILLDEHGHVR	Lys2	2.2
BRAF, BRAF V600E	P15056_V600E, P15056	DLKSNNIFLHEDLTVK	Lys2	0.5

Kinase	Reference	Sequence	Labeling Site	51-106 5 µM
BRAF, RAF1	P04049, P15056	IGDFGLATVKSR	Activation Loop	12.7
CaMK1a	Q14012	LVAIKCIAK	Lys1	NB
CaMK1d	Q8IU85	LFAVKCIPK	Lys1	30.3
CaMK1d	Q8IU85	DLKPENLLYYSQDEESK	Lys2	NB
CaMK2a, CaMK2b, CaMK2d, CaMK2g	Q13555, Q53H78, Q9UQM7, Q13557	DLKPENLLLASK	Lys2	NB
CaMK2d	Q13557	IPTGQEYAAKIINTKK	Lys1	NB
CaMK2g	Q13555	TSTQEYAAKIINTK	Lys1	7.1
CaMKK1	Q8N5S9	DIKPSNLLLGDDGHVK	Lys2	NB
CaMKK2	Q96RR4	LAYNENDNTYYAMKVLSK	Lys1	2.6
CaMKK2	Q96RR4	DIKPSNLLVGEDGHIK	Lys2	NB
CASK	O14936	ETGQQFAVKIVDVAK	Lys1	NB
CCRK	Q8IZL9	DLKPANLLISASGQLK	Lys2	4.5
CDC2	Q5H9N4	TTGQVVAMKK	Lys1	20.3
CDC2	Q5H9N4	DLKPQNLLIDDK	Lys2	NB
CDC2	Q5H9N4	KPLFHGDSEIDQLFR	Protein Kinase Domain	NB
CDK10	Q15131	DLKVSNLLMTDK	Lys2	10.3
CDK11	Q9BWU1	EYALKQIEGTGISMSACR	Lys1	4.0
CDK11, CDK8	P49336, Q9BWU1	DLKPANILVMGEGPER	Lys2	15.4
CDK2	P24941	LTGEVVALKK	Lys1	12.3
CDK2	P24941	DLKPQNLLINTEGAIK	Lys2	NB
CDK4	P11802	DLKPENILVTSGGTVK	Lys2	3.2
CDK5	Q00535	DLKPQNLLINR	Lys2	6.5
CDK5	Q00535	NRETHEIVALKR	Lys1	NB
CDK6	Q00534	DLKPQNILVTSSGQIK	Lys2	NB
CDK7	P50613	DKNTNQIVAIKK	Lys1	16.6

Kinase	Reference	Sequence	Labeling Site	51-106 5 µM
CDK7	P50613	DLKPNNLLLDENGVLK	Lys2	1.6
CDK9	P50750	IGQGTFGEVFKAR	ATP Loop	NB
CDK9	P50750	DMKAANVLITR	Lys2	NB
CHED	Q14004	DIKCSNILLNNR	Lys2	NB
CHK1	B4DT73	DIKPENLLLDER	Lys2	NB
CHK2	O96017	VAIKIISK	Lys1	14.8
CHK2	O96017	DLKPENVLLSSQEEDCLIK	Lys2	NB
CK1a	P48729	DIKPDNFLMGIGR	Lys2	11.1
CK1d, CK1e	P49674, P48730	DVKPDNFLMGLGKK	Lys2	NB
CK1g1	Q9HCP0	DVKPENFLIGR	Lys2	NB
CK1g1, CK1g2, CK1g3	Q9Y6M4, P78368, Q9HCP0	KIGCGNFGELR	ATP Loop	11.7
CK1g2	P78368	DVKPENFLVGRPGTK	Lys2	NB
CK1g3	Q9Y6M4	DVKPENFLIGRPGNK	Lys2	11.5
CK2a1	P68400	GGPNIITLADIVKDPVSR	Protein Kinase Domain	NB
CLK1	P49759	LTHTDLKPENILFVQSDYTEAYNPK	Lys2	0.5
CLK2	P49760	LTHTDLKPENILFVNSDYELTYNLEK	Lys2	NB
CLK3	P49761	FLHENQLTHTDLKPENILFVNSEFETLYNEHK	Lys2	4.4
CLK3	P49761	YEIVGNLGEGTFGKVVECLDHAR	ATP Loop	NB
CRK7	Q9NYV4	DIKCSNILLNNSGQIK	Lys2	14.3
CSK	P41240	VAVKCIK	Lys1	NB
CSK	P41240	VSDFGLTKEASSTQDTGKLPVK	ATP Loop	NB
DCAMKL2	Q8N568	DIKPENLLVCEYPDGTK	Lys2	NB
DGKA	P23743	IDPVPNTHPLLVFVNPKSGGK	ATP	NB
DGKH	Q86XP1	ATFSFCVSPLLVFVNSKSGDNQGVK	ATP	NB
DLK	Q12852	DLKSPNMLITYDDVVK	Lys2	5.9

Kinase	Reference	Sequence	Labeling Site	51-106 5 μM
DNAPK	P78527	KGGSWIQEINVAEK	ATP	NB
DNAPK	P78527	EHPFLVKGGEDLR	ATP	NB
DRAK1	Q9UEE5	DVVHLDLKPQNILLTSESPLGDIK	Lys2	NB
DRAK1, DRAK2	Q9UEE5, O94768	GKFAVVR	ATP Loop	4.6
eEF2K	O00418	YIKYNSNSGFVR	ATP	6.3
EGFR, EGFR-L858R	P00533-L858R, P00533	IPVAIKELR	Lys1	5.5
EGFR, EGFR-L858R	P00533-L858R, P00533	LLGAEEKEYHAEGGKVPIK	ATP Loop	NB
EphA2	P29317	VLEDDPEATYTTSGGKIPIR	ATP Loop	NB
EphB2	P29323	FLEDDTSDPTYTSALGGKIPIR	ATP Loop	NB
EphB3	P54753	FLEDDPSDPTYTSSLGGKIPIR	ATP Loop	1.6
EphB4	P54760	FLEENSSDPTYTSSLGGKIPIR	ATP Loop	1.6
Erk1	P27361	DLKPSNLLINTTCDLK	Lys2	NB
Erk2	P28482	DLKPSNLLLNTTCDLK	Lys2	NB
Erk3	Q16659	DLKPANLFINTEDLVLK	Lys2	NB
Erk5	Q13164	DLKPSNLLVNENCELK	Lys2	11.6
FAK	Q05397	YMEDSTYYKASK	ATP Loop	22.5
FAK	Q05397	CIGEGQFGDVHQGIYMSPENPALAVAIKTCK	Lys1	2.8
FAM20B	O75063	ETEPACADGDIMEGSVTLWLPDVWPLQKHR	ATP	6.6
FER	P16591	TSVAVKTCKEDLPQELK	Lys1	NB
FER	P16591	QEDGGVYSSSGLKQIPIK	ATP Loop	NB
FGFR1, FMS, PDGFRb, TYRO3	Q06418, P11362, P07333, P09619	VAVKMLK	Lys1	NB
FRAP	P42345	IQSIAPSLQVITSKQRPR	ATP	NB
FRK	P42685	HEIKLPVK	ATP Loop	NB
FYN, SRC, YES	P07947, P06241, P12931	QGAKFPIKWTAPEAALYGR	ATP Loop	14.0

Kinase	Reference	Sequence	Labeling Site	51-106<br>5 μM
GAK	O14976	DLKVENLLLSNQGTIK	Lys2	1.0
GCK	Q12851, P35557	DTVTSELAAVKIVK	Lys1	18.3
GCK	Q12851, P35557	DIKGANLLLTLQGDVK	Lys2	3.9
GCN2 domain2	Q9P2K8	LDGCCYAVKR	Lys1	5.3
GCN2 domain2	Q9P2K8	DLKPVNIFLDSDDHVK	Lys2	NB
GSK3A	P49840	DIKPQNLLVDPDTAVLK	Lys2	NB
GSK3B	P49841	DIKPQNLLLDPDTAVLK	Lys2	NB
HCK	P08631	VAVKTMKPGSMSVEAFLAEANVMK	Lys1	NB
HCK	P08631	EGAKFPIKWTAPEAINFGSFTIK	ATP Loop	NB
HER2/ErbB2	P04626	GIWIPDGENVKIPVAIKVLR	Lys1	15.6
HER2/ErbB2	P04626	LLDIDETEYHADGGKVPIK	ATP Loop	NB
HER3/ErbB3	P21860	SPSQVQVADFGVADLLPPDDKQLLYSEAKTPIK	ATP Loop	2.0
HER3/ErbB3	P21860	GVWIPEGESIKIPVCIKVIEDK	Lys1	NB
HRI	Q9BQI3	IGDFGLACTDILQKNTDWTNR	ATP Loop	NB
IKKa	O15111	DLKPENIVLQDVGGK	Lys2	NB
IKKb	O14920	WHNQETGEQIAIKQCR	Lys1	0.1
IKKb	O14920	DLKPENIVLQQGEQR	Lys2	NB
IKKe, TBK1	Q14164, Q9UHD2	DIKPGNIMR	Lys2	10.6
ILK	Q13418	WQGNDIVVKVLK	Lys1	NB
ILK	Q13418	ISMADVKFSFQCPGR	Protein Kinase Domain	NB
IRAK1	P51617	AIQFLHQDSPSLIHGDIKSSNVLLDER	Lys2	NB
IRAK4	Q9NWZ3	DIKSANILLDEAFTAK	Lys2	1.2
IRAK4	Q9NWZ3	GYVNNTTVAVKK	Lys1	NB
IRE1	O75460	GMFDNRDVAVKR	Lys1	8.9
IRE1	O75460	DLKPHNILISMPNAHGK	Lys2	NB

Kinase	Reference	Sequence	Labeling Site	51-106 5 μM
ITPK1	Q13572	NFSAGTSDRESIFFNSHNVSKPESSSVLTELDKIEGV FERPSDEVIR	ATP Loop	NB
JAK1 domain1	P23458	QLASALSYLEDKDLVHGNVCTKNLLLAR	ATP Loop	NB
JAK1 domain2	P23458	YDPEGDNTGEQVAVKSLKPESGGNHIADLKK	Lys1	NB
JAK1 domain2	P23458	IGDFGLTKAIETDKEYYTVK	ATP Loop	NB
JAK3 domain2	P52333	YDPLGDNTGALVAVKQLQHSGPDQQR	Lys1	25.2
JNK1, JNK2, JNK3	P45984, P53779, P45983	DLKPSNIVVK	Lys2	NB
KSR1, KSR2	Q6VAB6, Q8IVT5	SKNVFYDNGK	ATP Loop	8.8
LATS1	O95835	ALYATKTLR	Lys1	NB
LATS1	O95835	DIKPDNILIDR	Lys2	NB
LATS2	Q9NRM7	DIKPDNILIDLDGHIK	Lys2	1.4
LATS2	Q9NRM7	VDTHALYAMKTLR	Lys1	NB
LKB1	Q15831	DIKPGNLLLTTGGTLK	Lys2	NB
LOK	O94804	DLKAGNVLMTLEGDIR	Lys2	NB
LOK	O94804	NKETGALAAAKVIETK	Lys1	NB
LYN	P07948	VAVKTLKPGTMSVQAFLEEANLMK	Lys1	1.8
LYN	P07948	EGAKFPIKWTAPEAINFGCFTIK	ATP Loop	NB
MAP2K1	Q02750	IMHRDVKPSNILVNSR	Lys2	11.0
MAP2K1, MAP2K2	Q02750, P36507	DVKPSNILVNSR	Lys2	1.2
MAP2K1, MAP2K2	Q02750, P36507	KLIHLEIKPAIR	Lys1	NB
MAP2K3	P46734	DVKPSNVLINK	Lys2	18.5
MAP2K3	P46734	HAQSGTIMAVKR	Lys1	NB
MAP2K3	P46734	MCDFGISGYLVDSVAKTMDAGCKPYMAPER	ATP Loop	NB
MAP2K4	P45985	LCDFGISGQLVDSIAKTR	ATP Loop	6.0
MAP2K4	P45985	DIKPSNILLDR	Lys2	NB
MAP2K5	Q13163	DVKPSNMLVNTR	Lys2	3.0

Kinase	Reference	Sequence	Labeling Site	51-106 5 µM
MAP2K6	L5KNH7, P52564	MCDFGISGYLVDSVAKTIDAGCKPYMAPER	ATP Loop	NB
MAP2K6	L5KNH7, P52564	DVKPSNVLINALGQVK	Lys2	NB
MAP2K6	L5KNH7, P52564	HVPSGQIMAVKR	Lys1	NB
MAP2K7	O14733	DVKPSNILLDER	Lys2	NB
MAP3K1	Q13233	DVKGANLLIDSTGQR	Lys2	78.1
MAP3K15, MAP3K5, MAP3K6	O95382, Q99683, Q6ZN16	IAIKEIPER	Lys1	NB
MAP3K2	Q9Y2U5	ELAVKQVQFDPDSPETSKEVNALECEIQLLK	Lys1	NB
MAP3K2, MAP3K3	Q9Y2U5, Q99759	DIKGANILR	Lys2	4.6
MAP3K3	Q99759	ELASKQVQFDPDSPETSKEVSALECEIQLLK	Lys1	11.4
MAP3K4	Q9Y6R4	VYTCISVDTGELMAMKEIR	Lys1	8.6
MAP3K4	Q9Y6R4	DIKGANIFLTSSGLIK	Lys2	7.1
MAP3K5	Q99683	DIKGDNVLINTYSGVLK	Lys2	11.7
MAP3K6	O95382	DIKGDNVLINTFSGLLK	Lys2	NB
MAP4K3	Q8IVH8	NVNTGELAAIKVIK	Lys1	4.7
MAP4K3	Q8IVH8	DIKGANILLTDNGHVK	Lys2	NB
MAP4K5	Q9Y4K4	NVHTGELAAVKIIK	Lys1	6.0
MAP4K5	Q9Y4K4	DIKGANILLTDHGDVK	Lys2	NB
MAPKAPK2, MAPKAPK3	P49137, Q16644	DVKPENLLYTSK	Lys2	NB
MAPKAPK3	Q16644	QVLGLGVNGKVLECFHR	ATP Loop	NB
MAPKAPK3	Q16644	CALKLLYDSPK	Lys1	NB
MARK1, MARK2	Q7KZI7, Q9P0L2	EVAVKIIDK	Lys1	NB
MARK2	Q7KZI7	EVAVKIIDKTQLNSSSLQK	Lys1	5.0
MARK2, MARK3	Q7KZI7, P27448	DLKAENLLLDADMNIK	Lys2	11.3
MARK3	P27448	EVAIKIIDKTQLNPTSLQK	Lys1	NB
MARK3, MARK4	P27448, Q96L34	EVAIKIIDK	Lys1	NB

MARK4	Q96L34	DLKAENLLLDAEANIK	Lys2	10.3

Kinase	Reference	Sequence	Labeling Site	51-106 5 µM
MARK4	Q96L34	EVAIKIIDKTQLNPSSLQK	Lys1	NB
MAST1, MAST2	Q6P0Q8, Q9Y2H9	DLKPDNLLITSMGHIK	Lys2	NB
MAST3	O60307	DLKPDNLLITSLGHIK	Lys2	NB
MAST4	O15021	DLKPDNLLVTSMGHIK	Lys2	NB
MASTL	Q96GX5	GAFGKVYLGQK	ATP Loop	8.6
MASTL	Q96GX5	LYAVKVVK	Lys1	NB
MELK	Q14680	DLKPENLLFDEYHK	Lys2	NB
MER, TYRO3	Q12866, Q06418	KIYSGDYYR	ATP Loop	NB
MET	P08581	DMYDKEYYSVHNK	ATP Loop	8.3
MLK1	P80192	DLKSSNILILQK	Lys2	1.5
MLK3	Q16584	DLKSNNILLLQPIESDDMEHK	Lys2	1.3
MLK4	Q5TCX8	DLKSSNILLLEK	Lys2	NB
MLKL	Q8NB16	APVAIKVFK	Lys1	NB
MPSK1	O75716	DLKPTNILLGDEGQPVLMDLGSMNQACIHVEGSR	Lys2	NB
MRCKb	Q9Y5S2	DIKPDNVLLDVNGHIR	Lys2	NB
MSK1 domain1	O75582	DIKLENILLDSNGHVVLTDFGLSK	Lys2	NB
MSK1 domain1, MSK2 domain1	O75582, O75676	VLGTGAYGKVFLVR	ATP Loop	0.1
MSK2 domain1	O75676	LYAMKVLR	Lys1	5.5
MSK2 domain1	O75676	DLKLENVLLDSEGHIVLTDFGLSK	Lys2	NB
MST1	Q13043	ETGQIVAIKQVPVESDLQEIIK	Lys1	NB
MST1, MST2	Q13188, Q13043	LADFGVAGQLTDTMAKR	ATP Loop	2.7
MST1, MST2	Q13188, Q13043	DIKAGNILLNTEGHAK	Lys2	NB
MST2	Q13188	ESGQVVAIKQVPVESDLQEIIK	Lys1	0.8
MST3	Q9Y6E0	DIKAANVLLSEHGEVK	Lys2	4.2

MST3, MST4, YSK1	Q9Y6E0, O00506, Q9P289	LADFGVAGQLTDTQIKR	ATP Loop	NB

Kinase	Reference	Sequence	Labeling Site	51-106 5 µM
MST4, YSK1	O00506, Q9P289	DIKAANVLLSEQGDVK	Lys2	12.8
NDR1	Q15208	DTGHVYAMKILR	Lys1	6.0
NDR1	Q15208	DIKPDNLLLDSK	Lys2	NB
NDR1, NDR2	Q9Y2H1, Q15208	LSDFGLCTGLKK	ATP Loop	NB
NDR2	Q9Y2H1	DIKPDNLLLDAK	Lys2	7.6
NDR2	Q9Y2H1	DTGHIYAMKILR	Lys1	NB
NEK1	Q96PY6	DIKSQNIFLTK	Lys2	NB
NEK1	Q96PY6	QYVIKEINISR	Lys1	NB
NEK2	P51955	DLKPANVFLDGK	Lys2	NB
NEK3	P51956	SKNIFLTQNGK	ATP Loop	5.1
NEK4	P51957	DLKTQNVFLTR	Lys2	5.8
NEK6, NEK7	Q9HC98, Q8TDX7	DIKPANVFITATGVVK	Lys2	24.8
NEK7	Q8TDX7	AACLLDGVPVALKK	Lys1	10.3
NEK8	Q86SG6	DLKTQNILLDK	Lys2	NB
NEK9	Q8TD19	DIKTLNIFLTK	Lys2	13.8
NEK9	Q8TD19	RTEDDSLVVWKEVDLTR	Lys1	0.6
NEK9	Q8TD19	LGDYGLAKK	ATP Loop	NB
NLK	Q9UBE8	DIKPGNLLVNSNCVLK	Lys2	5.9
NuaK1	O60285	VVAIKSIR	Lys1	NB
NuaK2	Q9H093	LVAIKSIR	Lys1	2.2
p38a	Q16539	QELNKTIWEVPER	Other	7.5
p38a	Q16539	DLKPSNLAVNEDCELK	Lys2	NB
p38b	Q15759	QELNKTVWEVPQR	Other	NB
p38d, p38g	P53778, O15264	DLKPGNLAVNEDCELK	Lys2	NB

p70S6K	P23443	DLKPENIMLNHQGHVK	Lys2	NB
p70S6Kb	Q9UBS0	DLKPENIMLSSQGHIK	Lys2	22.5

Kinase	Reference	Sequence	Labeling Site	51-106 5 µM
p70S6Kb	Q9UBS0	IYAMKVLR	Lys1	22.5
PAK2	Q13177	IGQGASGTVFTATDVALGQEVAIKQINLQK	Lys1	6.9
PAK2	Q13177	KNPQAVLDVLKFYDSNTVK	Other	0.8
PAN3	Q58A45	VMDPTKILITGK	ATP	4.4
PAN3	Q58A45	IQKSSNFGYITSCYK	ATP	NB
PCTAIRE1	Q00536	SKLTDNLVALKEIR	Lys1	NB
PCTAIRE1, PCTAIRE3	Q07002, Q00536	DLKPQNLLINER	Lys2	4.8
PCTAIRE2	Q00537	DLKPQNLLINEK	Lys2	9.7
PCTAIRE2, PCTAIRE3	Q07002, Q00537	SKLTENLVALKEIR	Lys1	NB
PDK1	O15530	EYAIKILEK	Lys1	NB
PEK	Q9NZJ5	DLKPSNIFFTMDDVVK	Lys2	10.9
PFTAIRE1	O94921	LVALKVIR	Lys1	7.7
PFTAIRE2	Q96Q40	DLKPQNLLISHLGELK	Lys2	NB
PHKg2	P15735	ATGHEFAVKIMEVTAER	Lys1	NB
PI4K2B	Q8TCG2	SEEPYGQLNPKWTK	ATP	6.4
PI4KA, PI4KAP2	P42356, A4QPH2	SGTPMQSAAKAPYLAK	ATP	3.0
PI4KB	Q9UBF8	VPHTQAVVLNSKDK	ATP	3.9
PI4KB	Q9UBF8	LLSVIVKCGDDLRQELLAFQVLK	ATP	NB
PIK3C2B	O00750	VIFKCGDDLRQDMLTLQMIR	ATP	2.9
PIK3C3	Q8NEB9	TEDGGKYPVIFKHGDDLR	ATP	4.9
PIK3C3	Q8NEB9	TEDGGKYPVIFKHGDDLRQDQLILQIISLMDK	ATP	NB
PIK3CA	P42336	IMSSAKRPLWLNWENPDIMSELLFQNNEIIFK	ATP	NB
PIK3CB	P42338	VFGEDSVGVIFKNGDDLRQDMLTLQMLR	ATP	31.4
PIK3CB	P42338	VFGEDSVGVIFKNGDDLR	ATP	1.7

PIK3CD	O00329	VNWLAHNVSKDNRQ	ATP	1.8
PIP4K2A	P48426	YIIKTITSEDVAEMHNILK	ATP	4.5

Kinase	Reference	Sequence	Labeling Site	51-106 5 μM
PIP4K2A	P48426	AKELPTLKDNDFINEGQK	ATP	NB
PIP4K2B	P78356	AKDLPTFKDNDFLNEGQK	ATP	NB
PIP4K2C	Q8TBX8	TLVIKEVSSEDIADMHSNLSNYHQYIVK	ATP	21.8
PIP4K2C	Q8TBX8	VKELPTLKDMDFLNK	ATP	NB
PIP5K1A	Q99755	EKPLPTFKDLDFLQDIPDGLFLDADMYNALCK	ATP	8.7
PIP5K3	Q9Y2I7	GGKSGAAFYATEDDRFILK	ATP	NB
PITSLRE	P21127	DLKTSNLLLSHAGILK	Lys2	NB
PKCa, PKCb	P05771, P17252	DLKLDNVMLDSEGHIK	Lys2	NB
PKCa, PKCg	P17252, P05129	NLIPMDPNGLSDPYVKLK	Other	NB
PKCd	Q05655	DLKLDNVLLDR	Lys2	0.4
PKCd	Q05655	KPTMYPEWK	Other	NB
PKCi	P41743	IYAMKVVK	Lys1	NB
PKCi	P41743	DLKLDNVLLDSEGHIK	Lys2	NB
PKD1	Q15139	DVAIKIIDK	Lys1	NB
PKD1, PKD2	Q9BZL6, Q15139	NIVHCDLKPENVLLASADPFPQVK	Lys2	NB
PKD2	Q9BZL6	DVAVKVIDK	Lys1	10.6
PKD3	O94806	DVAIKVIDK	Lys1	27.0
PKD3	O94806	NIVHCDLKPENVLLASAEPFPQVK	Lys2	NB
PKN1	Q16512	DLKLDNLLLDTEGYVK	Lys2	14.3
PKN1	Q16512	VLLSEFRPSGELFAIKALK	Lys1	NB
PKN2	Q16513	DLKLDNLLLDTEGFVK	Lys2	3.7
PKR	P19525	DLKPSNIFLVDTK	Lys2	18.7
PKR	P19525	IGDFGLVTSLKNDGKR	ATP Loop	NB
PLK1	P53350	DLKLGNLFLNEDLEVK	Lys2	10.9

PLK1	P53350	CFEISDADTKEVFAGKIVPK	Lys1	NB
PLK2	Q9NYY3	DLKLGNFFINEAMELK	Lys2	1.4

**Kinase**	**Reference**	**Sequence**	**Labeling Site**	**51-106 5 µM**
PRP4	Q13523	CNILHADIKPDNILVNESK	Lys2	NB
QSK	Q9Y2K2	VAIKIIDKTQLDEENLKK	Lys1	NB
RAF1	P04049	DMKSNNIFLHEGLTVK	Lys2	NB
RIPK1	Q13546	DLKPENILVDNDFHIK	Lys2	3.7
RIPK2	O43353	ILHEIALGVNYLHNMTPPLLHHDLKTQNILLDNEFHVK	Lys2	NB
ROCK1	Q13464	KLQLELNQER	Other	NB
ROCK1, ROCK2	Q13464, O75116	VYAMKLLSK	Lys1	11.9
ROCK1, ROCK2	Q13464, O75116	DVKPDNMLLDK	Lys2	NB
RON	Q04912	IQCAIKSLSR	Lys1	NB
RSK1 domain1	Q15418	LTDFGLSKEAIDHEKK	ATP Loop	NB
RSK1 domain1, RSK2 domain1, RSK3 domain1	P51812, Q15349, Q15418	DLKPENILLDEEGHIK	Lys2	NB
RSK1 domain2	Q15418	DLKPSNILYVDESGNPECLR	Lys2	6.9
RSK2 domain1	P51812	QLYAMKVLK	Lys1	23.6
RSK2 domain1	P51812	LTDFGLSKESIDHEKK	ATP Loop	NB
RSK2 domain2	P51812	DLKPSNILYVDESGNPESIR	Lys2	5.7
RSK3 domain1	Q15349	DLKPENILLDEEGHIKITDFGLSK	Lys2	NB
SBK	Q52WX2	DIKPENVLLFDR	Lys2	NB
SGK3	Q96BR1	FYAVKVLQK	Lys1	NB
SIK	P57059	TQVAIKIIDK	Lys1	23.3
SLK	Q9H2G2	DLKAGNILFTLDGDIK	Lys2	7.0
SLK	Q9H2G2	AQNKETSVLAAAKVIDTK	Lys1	NB
SMG1	Q96Q15	SYPYLFKGLEDLHLDER	ATP	NB
SMG1	Q96Q15	DTVTIHSVGGTITILPTKTKPK	ATP	NB

SNRK	Q9NRH2	DLKPENVVFFEK	Lys2	0.4
SNRK	Q9NRH2	VAVKVIDK	Lys1	NB
SRPK1	Q96SB4	IIHTDIKPENILLSVNEQYIR	Lys2	4.4

Kinase	Reference	Sequence	Labeling Site	51-106 5 μM
SRPK1, SRPK2	P78362, Q96SB4	FVAMKVVK	Lys1	NB
SRPK2	P78362	IIHTDIKPENILMCVDDAYVR	Lys2	NB
STK33	Q9BYT3	DLKLENIMVK	Lys2	26.6
STLK5	Q7RTN6	YSVKVLPWLSPEVLQQNLQGYDAK	ATP Loop	NB
STLK5	Q7RTN6	SVKASHILISVDGK	Lys2	NB
STLK6	Q9C0K7	HTPTGTLVTIKITNLENCNEER	Lys1	5.8
TAK1	O43318	DLKPPNLLLVAGGTVLK	Lys2	NB
TAO1, TAO3	Q7L7X3, Q9H2K8	DIKAGNILLTEPGQVK	Lys2	4.9
TAO2	Q9UL54	DVKAGNILLSEPGLVK	Lys2	5.5
TBK1	Q9UHD2	TGDLFAIKVFNNISFLRPVDVQMR	Lys1	5.7
TEC	P42680	YVLDDQYTSSSGAKFPVK	ATP Loop	20.1
TGFbR2	P37173	DLKSSNILVK	Lys2	8.9
TLK1	Q9UKI8	YLNEIKPPIIHYDLKPGNILLVDGTACGEIK	Lys2	6.2
TLK2	Q86UE8	YLNEIKPPIIHYDLKPGNILLVNGTACGEIK	Lys2	1.7
TLK2	Q86UE8	YVAVKIHQLNK	Lys1	1.2
TNK1	Q13470	SVPVAVKSLR	Lys1	1.9
TYK2 domain2	P29597,	IGDFGLAKAVPEGHEYYR	ATP Loop	NB
ULK1	O75385	DLKPQNILLSNPAGR	Lys2	NB
ULK3	D3DW67	EVVAIKCVAK	Lys1	NB
Wee1	P30291	YIHSMSLVHMDIKPSNIFISR	Lys2	2.2
Wnk1, Wnk2	Q9Y3S1, Q9H4A3	GSFKTVYK	ATP Loop	NB
Wnk1, Wnk2, Wnk3	Q9BYP7, Q9Y3S1, Q9H4A3	DLKCDNIFITGPTGSVK	Lys2	10.6

Wnk1, Wnk2, Wnk4	Q9Y3S1, Q96J92, Q9H4A3	IGDLGLATLKR	ATP Loop	4.6
YANK3	Q86UX6	DVKPDNILLDER	Lys2	NB
YES	P07947	VAIKTLKPGTMMPEAFLQEAQIMK	Lys1	16.0
ZAK	Q9NYL2	WISQDKEVAVKK	Lys1	NB
ZC1/HGK	O95819	TGQLAAIKVMDVTEDEEEEIKLEINMLKK	Lys1	3.1
ZC1/HGK, ZC2/TNIK, ZC3/MINK	Q9UKE5, O95819, Q8N4C8	DIKGQNVLLTENAEVK	Lys2	NB
ZC2/TNIK	Q9UKE5	TGQLAAIKVMDVTGDEEEEIKQEINMLKK	Lys1	11.2

51-106, 1H NMR DMSO-d6

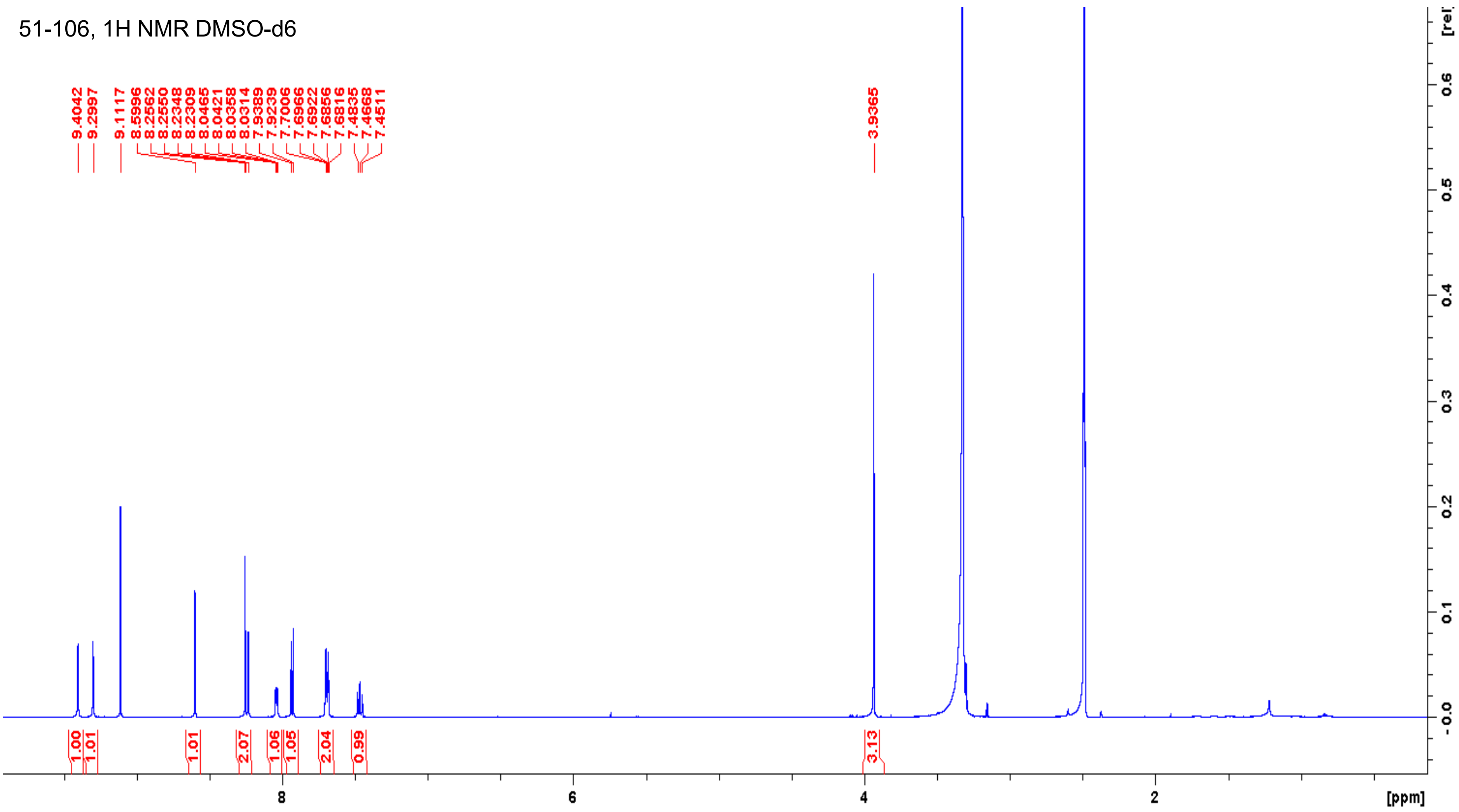

51-106, 1H NMR DMSO-d6

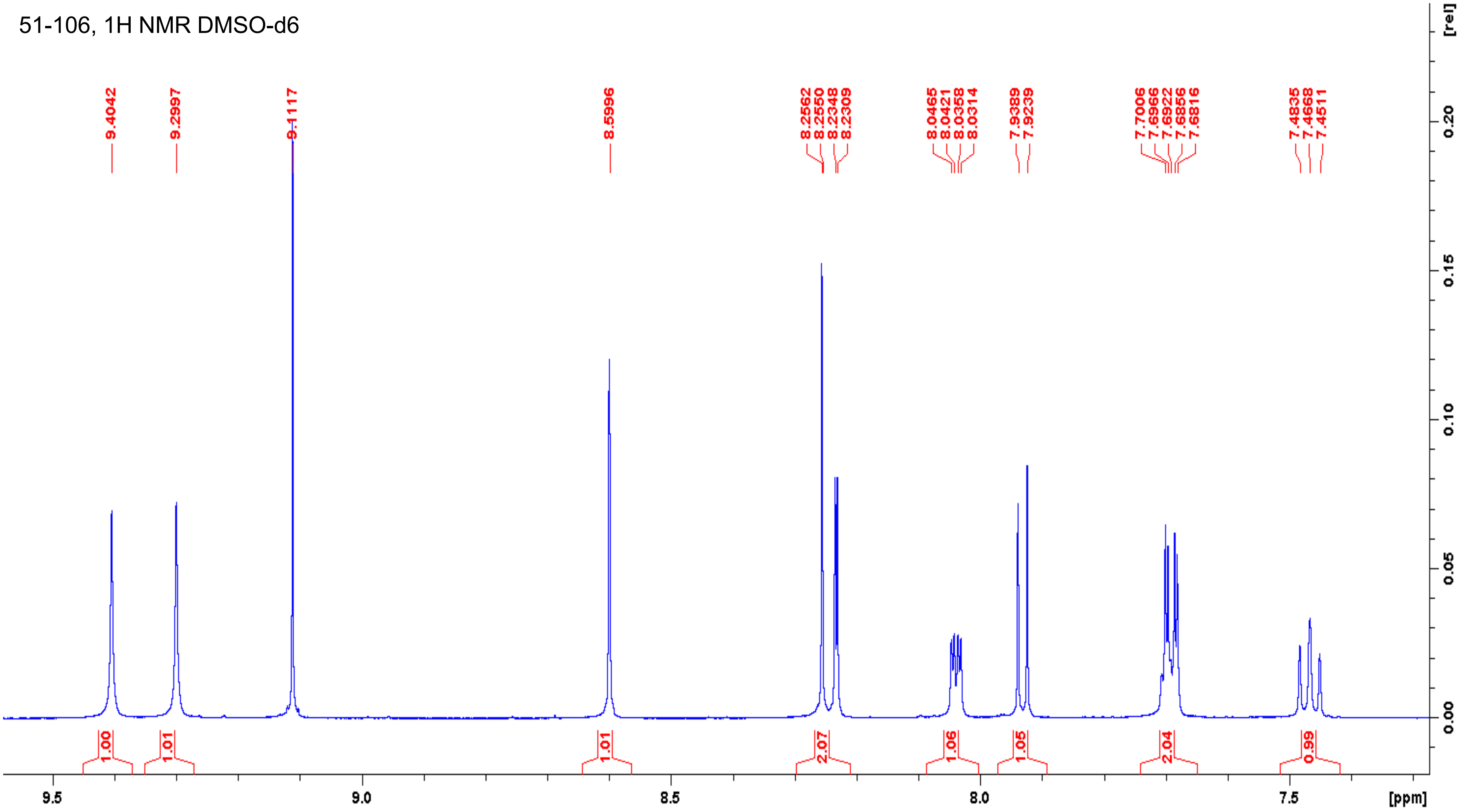

51-106, 13C NMR DMSO-d6

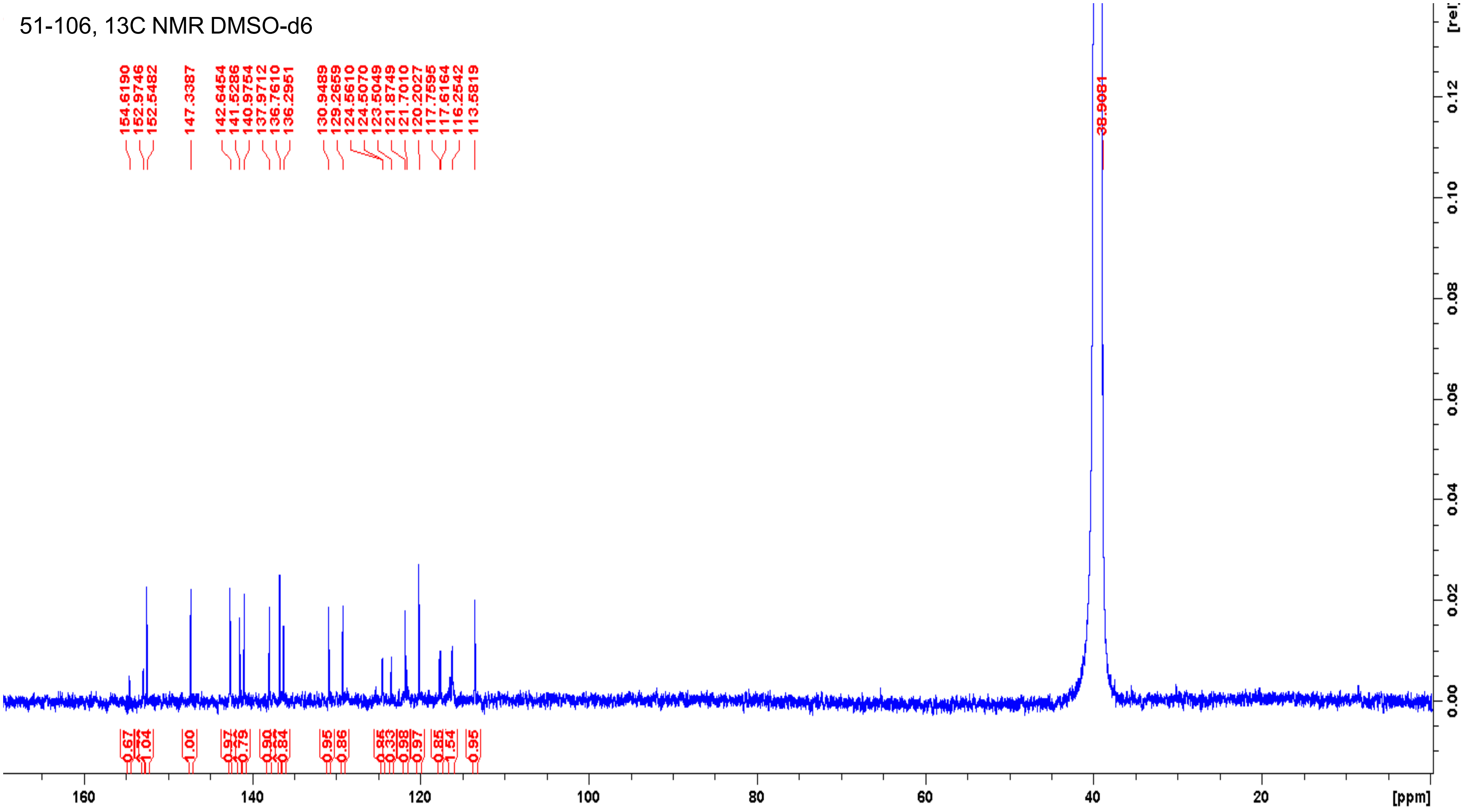

51-106, 13C NMR DMSO-d6

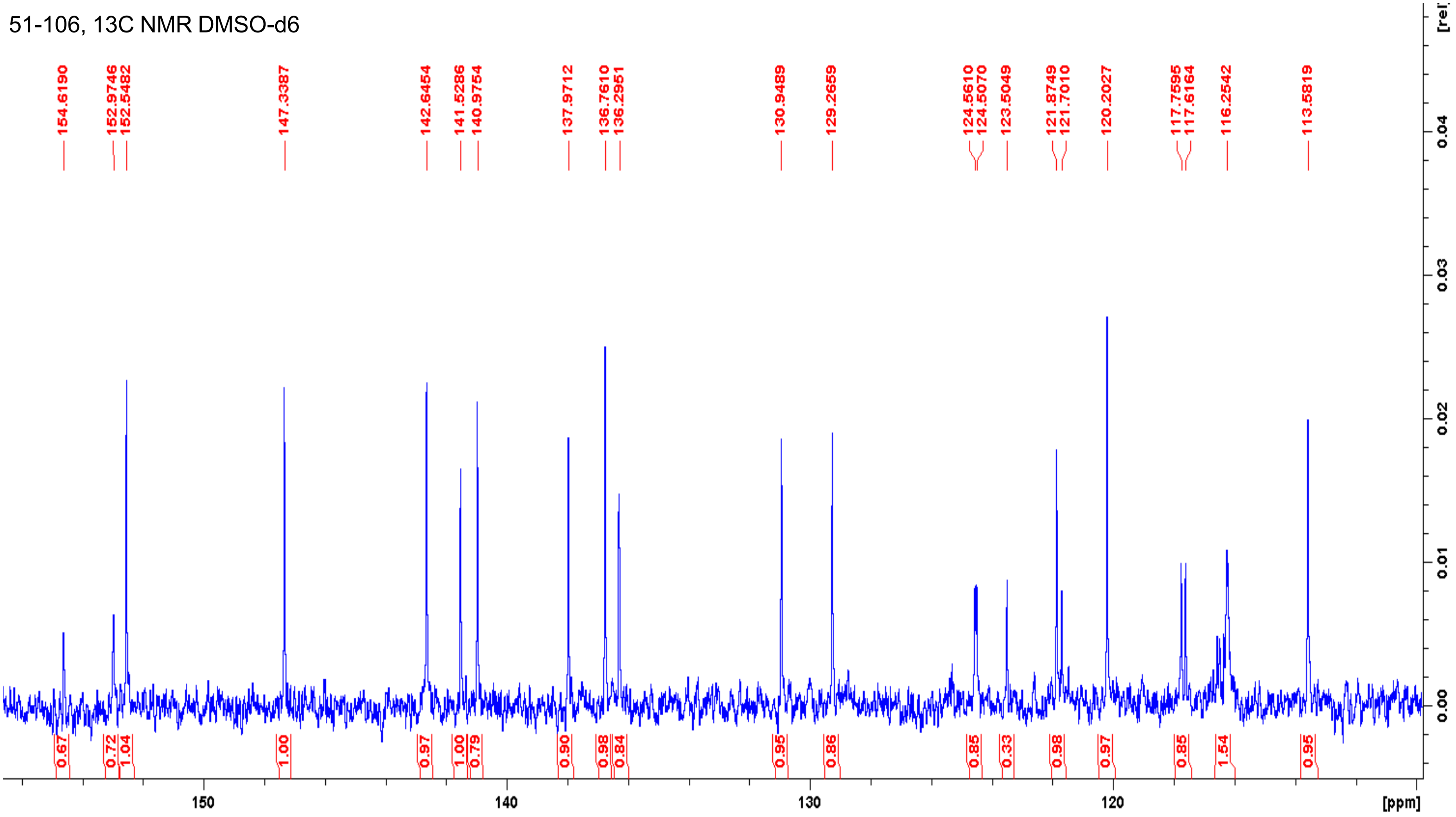

51-106, 19F NMR DMSO-d6

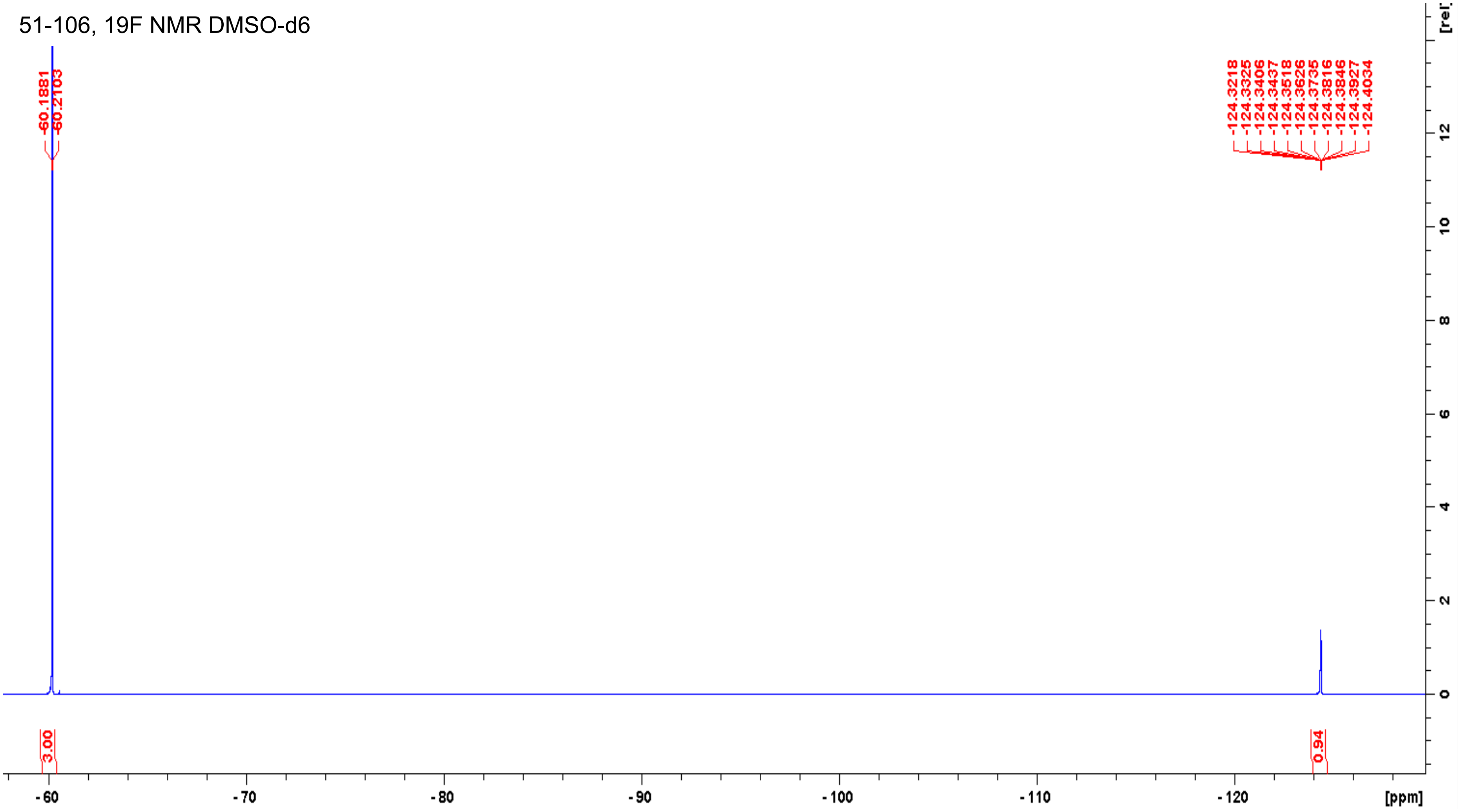

LC-MS from an Agilent 6230 LC/TOF mass spectrometer with an ESI source coupled with an Agilent Infinity 1260 HPLC system running in reverse phase on a ZORBAX Eclipse Plus C8 (4.6 × 150 mm, 3.5 μm) using water with 0.1% formic acid as solvent A and acetonitrile with 0.1% formic acid as solvent B. For analytical HPLC, the gradient for elution varied from 5% B to 95% B over 9 minutes and kept at this gradient for 3 minutes at a flow rate of 1.0 mL/min for a 12 min run.

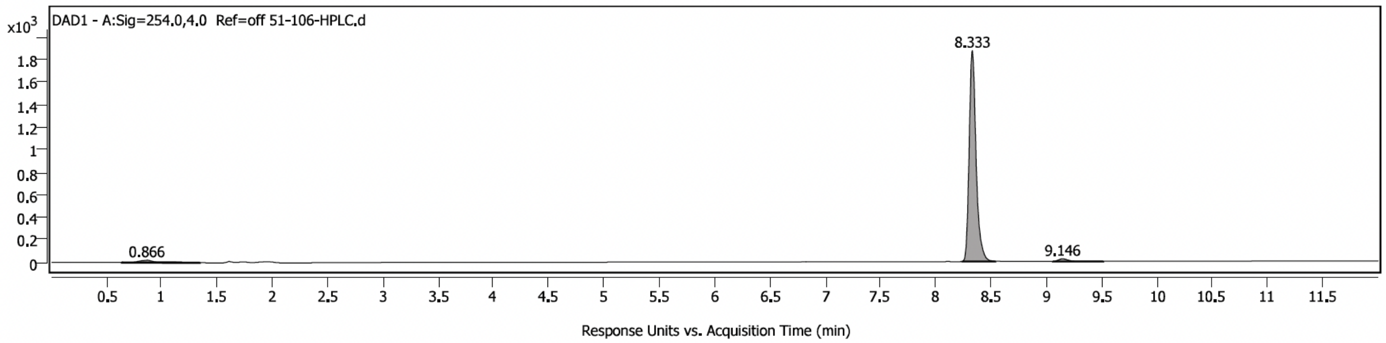

+ Scan (rt: 8.3 min)

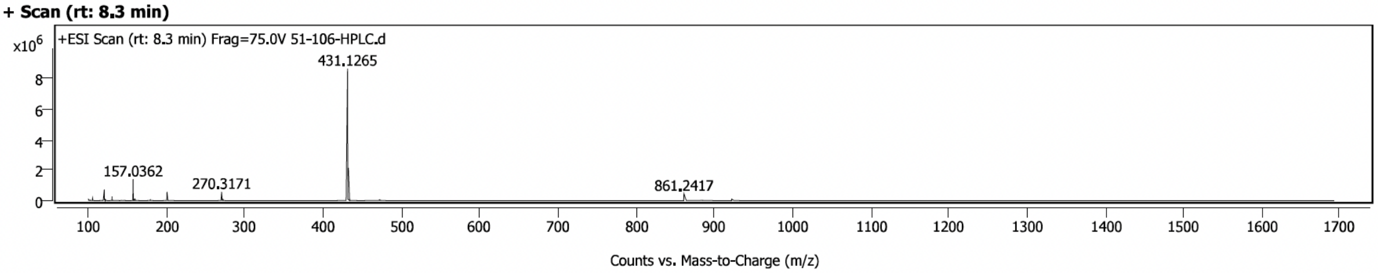

Supplement: Supplementary file 1 [file molecules-30-02001-s001.zip › molecules-3571308-supplementary.pdf]
